# The chiisanoside derivatives present in the leaves of *Acanthopanax sessiliflorus* activate autophagy through the LRP6/GSK3β axis and thereafter inhibit oxidative stress, thereby counteracting cisplatin-induced ototoxicity

**DOI:** 10.3389/fphar.2024.1518810

**Published:** 2025-01-15

**Authors:** Wenxin Zhang, Hongbo Teng, Tianyi Zhao, Roberts I. Eglitis, Xv Wang, Zhengxuan Yu, Shurong Qu, Haijing Wang, Yaru Zhao, Bowen Fan, Shuangli Liu, Yan Zhao

**Affiliations:** ^1^ College of Chinese Medicinal Materials, Jilin Agricultural University, Changchun, Jilin, China; ^2^ International Joint Laboratory for Development of Animal and Plant Resources for Food and Medicine, Jilin Agricultural University, Changchun, Jilin, China; ^3^ Institute of Changbai Mountain Biology Germplasm Resources, Tonghua Normal University, Tonghua, Jilin, China; ^4^ Institute of Solid State Physics, University of Latvia, Riga, Latvia

**Keywords:** cisplatin-induced ototoxicity (CIO), RNA sequencing, autophagy, oxidative stress, chiisanoside derivatives

## Abstract

**Introduction:**

Cisplatin is extensively employed in the treatment of multiple solid malignant tumors. Nevertheless, side effects such as cisplatin-induced ototoxicity (CIO) pose obstacles to tumor therapy.The important natural product chiisanoside from *Acanthopanax sessiliflorus* has abundant activity against CIO.

**Methods:**

In this study, 26 chiisanoside derivatives were screened, and compound 19 demonstrated significant protective activity against CIO damage. A cisplatin—induced HEI—OC1 cell injury model and a mouse ototoxicity model were established. The regulatory effects were revealed through transcriptome sequencing, and the protein expression levels were analyzed by molecular docking, ELISA, Western blotting, and immunofluorescence.

**Results:**

It was found that compound 19 inhibited cell apoptosis, alleviated abnormal hearing and spiral ganglion damage. Transcriptome sequencing revealed its regulatory effects. Compound 19 treatment increased autophagy levels, thereby alleviating mitochondrial dysfunction and reducing the accumulation of reactive oxygen species (ROS).In-depth studies have found that the autophagy inhibitor 3-methyladenine (3-MA) weakens the regulatory effect of compound 19 on autophagy and inhibits the clearance of damaged cells, resulting in oxidative stress damage, apoptosis and necrosis. By knocking down LRP6, it was found that the protective effect of compound 19 was eliminated, the autophagy level was significantly reduced, oxidative stress and ROS production were induced, and apoptosis after cisplatin exposure was promoted. Finally, the inhibitor LiCl was used to suppress the expression of GSK3β. It was found that inhibiting GSK3β could protect cells from cisplatin-induced damage by activating autophagy.

**Discussion:**

These findings suggest that compound 19 is capable of preventing ototoxicity by activating autophagy via the LRP6/GSK3β axis and consequently inhibiting oxidative stress, offering a new approach for treating CIO and sensorineural hearing loss.

## 1 Introduction

Hearing impairment ranks among the most common human afflictions, significantly impeding communication and diminishing the quality of life. According to data from the World Health Organization (WHO), more than 5% of the world’s population has a certain degree of hearing impairment (Gao et al., 2024). Genetic factors, aging, noise exposure, trauma, and ototoxic drugs are all causes of hearing impairment (Prasad et al., 2024). As one of the chemotherapy drugs, cisplatin has ototoxic side effects in about 62% of chemotherapy patients, usually manifested as progressive, bilateral, and irreversible hearing loss (Tang et al., 2021a). Research reports have stated that the characteristic of cisplatin-induced ototoxicity (CIO) is the loss of auditory hair cells due to apoptosis and necrosis, mainly damaging the organ of Corti, spiral ganglion, and stria vascularis (Yu et al., 2019; Anfuso et al., 2022; Adachi et al., 2023). At present, there is no definite method to prevent CIO. Possible hearing damage and its underlying mechanism during cisplatin chemotherapy have become a significant topic in otology research and have profoundly improved patients’ quality of life.

It is generally believed that oxidative stress is a key factor in the complex pathway leading to CIO damage. DNA cross-linking disrupts mitochondrial metabolism and damages the respiratory chain, resulting in changes in mitochondrial membrane permeability and potassium outflow. Therefore, reactive oxygen species (ROS) production is enhanced, ultimately leading to irreversible apoptosis (Marullo et al., 2013).

Autophagy is a lysosome-mediated degradation process of toxic proteins and unwanted organelles. Fundamentally, autophagy maintains the balance between organelle biogenesis, protein synthesis, and clearance (Kroemer et al., 2010). In hair cells, autophagy plays a crucial role in preventing ototoxicity. On the one hand, excessive activation of autophagy can promote apoptosis and the occurrence of pathological changes in HEI-OC1 cells (Li et al., 2018; Yin et al., 2018). On the other hand, autophagy can remove damaged organelles and has a protective effect on hair cell loss, spiral ganglion degeneration, and subsequent hearing impairment (Guo et al., 2021).

Low-density lipoprotein receptor-related protein 6 (LRP6) functions as a co-receptor within the Wnt/β-catenin signaling cascade and has been shown to dampen the activity of glycogen synthase kinase 3 beta (GSK3β) (Mi et al., 2006). Some studies have reported that lithium chloride (LiCl) inhibits the activity of GSK3β in models of cisplatin-induced acute kidney injury and ototoxicity (Park et al., 2009). Under physiological conditions, GSK3β inhibits the activation of autophagy. When cells are subjected to abnormal external stimuli, the upstream kinases in cells promote the phosphorylation and inactivation of GSK3β to block its inhibition of autophagy and restore intracellular homeostasis (Pan and Valapala, 2022). However, research on autophagy in the field of hearing is limited, and the role of LRP6/GSK3β in activating autophagy in cisplatin-induced ototoxicity (CIO) as well as the underlying molecular mechanisms remain unclear.

*Acanthopanax sessiliflorus* (Rupr. and Maxim.) Maxim., belonging to the Araliaceae family and the *Acanthopanax* genus. The roots, stems, leaves, and fruits of *Acanthopanax sessiliflorus* are all edible. There is a long-standing edible custom among the people in the producing area, and the edible history in some areas has been over 100 years long (Yun et al., 2023).Current scholarly research on *A. sessiliflorus* has predominantly centered on its roots and fruits, with comparatively less focus on the leaves (Lee et al., 2012; Kim G.-D. et al., 2024). In terms of pharmacological effects, the focus is mainly on its antioxidant and anti-inflammatory effects, and the research on other activities is still insufficient (Lee et al., 2015; Chen et al., 2020; Kim D. et al., 2024). Studies have shown that the active ingredients in the leaves of *A. sessiliflorus* can be used to treat various diseases such as arrhythmia, anti-tumor, and liver protection (Bian et al., 2019; Zhao et al., 2021; Wang et al., 2022; Teng et al., 2023), but there is currently no relevant report on its ototoxicity and hearing loss. The chiisanoside in *A. sessiliflorus* is known for its diverse pharmacological activities (Xiao et al., 2020). Our preliminary laboratory findings have indicated that chiisanoside, a secondary metabolite derived from the leaves of *A. sessiliflorus*, exhibits notable protective effects against CIO. In order to further optimize the clinical application of *A. sessiliflorus*, modifications are made based on the structure of chiisanoside to find candidate drugs with better activity to treat ototoxicity. Initially, we will conduct an activity screen of chiisanoside-related derivatives that have been synthesized in our lab. Subsequently, capitalizing on the pronounced protective effect of compound 19 in cisplatin-induced HEI-OC1 cells, we will employ RNA transcriptomics to probe the potential mechanisms and signaling pathways involved. Ultimately, it is verified by various molecular biology techniques, and the exact mechanism of action is elucidated at multiple levels such as molecules, cells, and animals.

This study reveals for the first time the important role of compound 19, a chiisanoside derivative from *A. sessiliflorus*, in alleviating CIO. We demonstrate that compound 19 potentially activates autophagy, thereby inhibiting oxidative stress and countering the detrimental effects of CIO. This is achieved by stimulating LRP6/GSK3β after cisplatin exposure. Our findings introduce compound 19 as a promising candidate for therapeutic intervention in CIO and sensorineural hearing loss, highlighting a new avenue for drug development and the identification of molecular targets within this context.

## 2 Materials and methods

### 2.1 Chemicals and reagents

The leaves of *A. sessiliflorus* were collected from the Medicinal Botanical Garden of Jilin Agricultural University and identified as *A. sessiliflorus* of the genus *Acanthopanax* in the family Araliaceae by Professor Zhao Yan. Cisplatin (CDDP) (Sigma-Aldrich, United States). DMEM medium (Gibco, China). FBS (Clark Bioscience, United States). Phosphate-buffered saline (PBS), trypsin, penicillin-streptomycin solution, 1% crystal violet staining solution, Trizol reagent, DAPI solution, BCA kit, RIPA lysis buffer, lactate dehydrogenase (LDH), FITC, TBST solution, tris-glycine SDS-PAGE electrophoresis buffer and transmembrane buffer (Solarbio, China). 3-methyladenine (3-MA), LiCl and rapamycin (RAP) (MCE, China). Fluorescein diacetate (FDA), propidium iodide (PI), Annexin V-FITC/PI kit (bestbio, China). Hoechst 33342 kit, JC-1 kit, Ros kit, loading buffer and hematoxylin-eosin (H&E) staining solution (Beyotime, China). Cell Counting Kit-8 (CCK-8), dimethyl sulfoxide (DMSO) (Sigma, United States). PVDF (Merck Millipore, Germany), 4% paraformaldehyde, skim milk powder, bovine serum albumin (BSA) and color pre-dyed protein Marker (Biosharp, China). Superoxide dismutase (SOD), malondialdehyde (MDA) and glutathione (GSH) kit (Nanjing Jiancheng Bioengineering Institute, China). Bax, Bcl-2 and Cleaved-Caspase-3 (Proteintech, China). Caspase-3 (Wanleibio, China). LC3II/I, P62, Atg7, Atg5, LRP6, GSK3β, P-GSK3β, TFEB, LaminB and β-actin (Abcam, United States). HRP-labeled goat anti-rabbit IgG and HRP-labeled rabbit anti-mouse IgG (Wanleibio, China). Sodium carboxymethyl cellulose (Aladdin, China). The LRP6 gene low-expression plasmid was purchased from Shanghai Heyuan Biotechnology Co., Ltd.

### 2.2 Preparation of test drugs

All the tested drugs were self-prepared in the laboratory with a purity of more than 95%. According to the previous research in the laboratory (Teng et al., 2023), 2 kg of dry leaves of *A*. *sessiliflorus* was extracted ultrasonically with a 75% ethanol solution. The ethanol extract was collected, filtered, and concentrated. Using D101 resin as the stationary phase, gradient elution was carried out with 10%, 30%, and 50% ethanol solutions to obtain the primary product of Chiisanoside (160 g, with a yield of 7%–8%). Elution was carried out with 100–200 mesh and 200–300 mesh silica gel, using chloroform/methanol (6:1/3:1) as the mobile phase. The 3:1 component was collected and evaporated to dryness to obtain pure chiisanoside (120 g, with a yield of 75%).

Chiisanoside (955 mg, 1 mmol) was dissolved in a 10% NaOH methanol solution and heated at 95°C for 2 h. Then, the methanol solvent was removed under reduced pressure. After that, 3 mol/mL hydrochloric acid was added for 4 h. Next, the solvent after the reaction was filtered under reduced pressure to obtain a white powder. After elution with ultrapure water until nearly neutral, it was evaporated to dryness to obtain a by-product of chiisanogenin. Using 300–400 mesh silica gel as the stationary phase, elution and separation (chloroform/methanol = 50:1/10:1) were performed to obtain pure chiisanogenin.

Chiisanoside (955 mg, 1 mmol) was dissolved in the mixed solution of hydrochloric acid and methanol/ethanol/n-propanol/n-butanol (3 mol/L hydrochloric acid solution), and heated under reflux for 4–6 h. Then NaOH solution was added to adjust the pH value of the reaction solvent to neutrality. Subsequently, separation was carried out using 300–400 mesh silica gel (chloroform/methanol = 60:1/30:1) to obtain compounds SC, 11, 17, and 23.

Chiisanogenin; compounds SC, 11, and 17 (1.0 mmol); and coupling catalysts EDC (4.0 mmol) and HOBt (4.0 mmol) were first dissolved in DMF (mass ratio = 1∶20), followed by the addition of 2-thiophene ethylamine (4.0 mmol)/2-ethoxyethylamine (4.0 mmol)/Boc-guanidine (4.0 mmol)/triethylamine (4.0 mmol)/diethanolamine (4.0 mmol) and a small amount of 4 A molecular sieves. The mixture was refluxed at 90°C for 24 h. The solvent was then evaporated under reduced pressure. Gradient elution and purification were performed using silica gel (300–400 mesh) with a solvent system of chloroform/methanol (70:1/30:1) to obtain compounds 1–5, compounds 6–10, compounds 12–16, and compounds 18–22.

**FIGURE 1:**
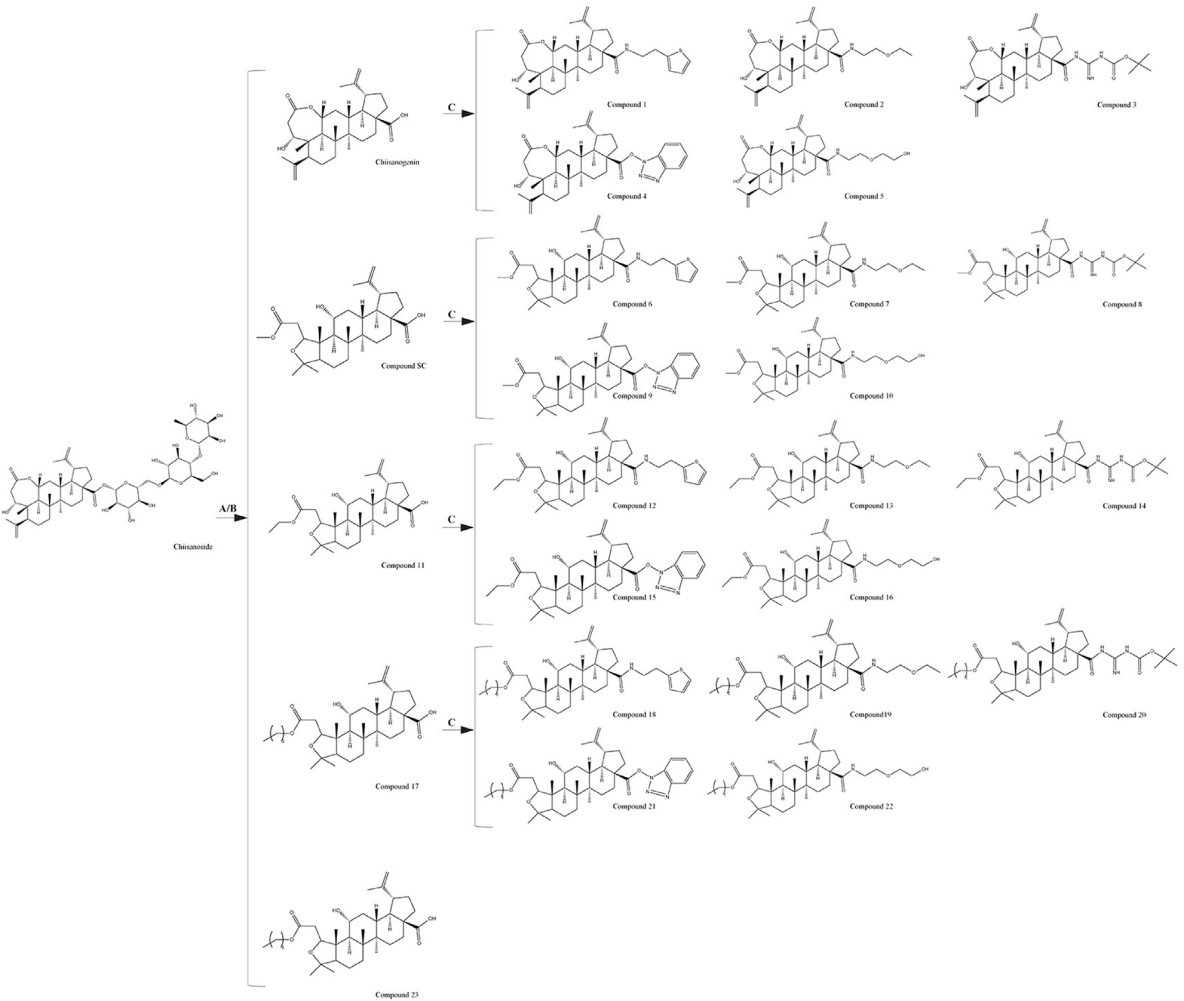
Synthesis methods of compounds 1 - 23, **(A)** 10% NaOH, MeOH, Reflux, 4 h, HCl, Stir, 2 h; **(B)** HCl (3 mol/L), MeOH/EtOH/Pro-OH/But-OH, Reflux, 2 h; **(C)** DMF, EDCI, HOBT, 4A molecular sieve, heating, 24 h.

The chemical formulas and synthesis methods of all the tested drugs can be found in Figure 1. For detailed information on structural identification, please refer to the reference (Teng et al., 2023) in detail. The structural identification related to compound 19 is shown in Supplementary Figures S1, S2. All test drugs were prepared using 100 mM dimethyl sulfoxide as the solvent.

### 2.3 Cell culture and treatment and cell viability assay

The mouse cochlear hair cell line (HEI-OC1) was provided by the Third Hospital of Jilin University. HepG2 human liver cancer cells, MCF-7 human breast cancer cells, and T24 human bladder cancer cells were all purchased from the Cell Bank of the Chinese Academy of Sciences (GNM25).

Investigate the effects of different compounds at different concentrations on the viability of HEI-OC1 cells: Cells were treated with compounds at concentrations of 6.25, 12.5, 25, 50, and 100 μM for 24 h, and a blank control well was set up. 10 μL of CCK-8 solution was added to each well and incubated in an incubator for 2 h. The absorbance was measured using a microplate reader (SpectraMax 190; Molecular Devices Co., Ltd.) and calculated using GraphPad Prism 6.0 software.

Explore the effects of different concentrations of cisplatin on the viability of HEI-OC1 cells: Cells were treated with cisplatin at concentrations of 6.25, 12.5, 25, 50, and 100 μM for 24 h, and a control group was included. The CCK-8 method was used to assess the cell viability.

Investigate the protective effects of different compounds at various concentrations against cisplatin-induced injury in HEI-OC1 cells: Cells were treated with compounds at concentrations of 6.25, 12.5, and 25 μM for 2 h, and then treated with 50 μM cisplatin for 24 h. The CCK-8 method was used to assess cell viability.

Divide the HEI-OC1 cells into a control group, a CDDP group, a CDDP + compound 19 (6.25 μM) group, a CDDP + compound 19 (12.5 μM) group, and a CDDP + compound 19 (25 μM) group. The control group received no treatment. Model cells were cultured in medium containing CDDP (50 μM) for 24 h. The administration group was given compound 19 at different concentrations first, and after 2 h, CDDP was added and cultured for another 24 h. To evaluate the protective effect of autophagy, the autophagy inducer rapamycin (RAP) and the autophagy inhibitor 3-MA were co-administered with the compound 19. The groups treated with these agents were named as follows: CDDP + 3-MA (5 mM) group, CDDP + RAP (0.1 μM) group, and CDDP + compound 19 + 3-MA group. To verify the effect of GSK3β on cells, the GSK3β inhibitor LiCl (10 μM) was administered.

The effect of compound 19 and cisplatin on the cell viability of human liver cancer cells: HepG2 cells were cultured using routine methods and divided into a control group, a CDDP group (10 μM, determined based on experimental exploration), and a compound 19 (25 µM) + CDDP group. After co-treating the cells, cell viability was measured using the CCK-8 assay 24 h later.

The effect of compound 19 and cisplatin on the cell viability of human breast cancer cells: MCF-7 cells were cultured using routine methods and treated with compound 19 (25 µM) in combination with cisplatin (8 μM, determined based on experimental exploration); a CDDP group was induced to cause cell damage with cisplatin; and a control group was set up. Cell viability was measured 24 h later.

The effect of compound 19 and cisplatin on the cell viability of human bladder cancer cells: T24 cells were cultured using routine methods and treated with cisplatin (5 μM, determined based on experimental exploration) and compound 19 (25 µM) in combination with cisplatin; a control group was also set up. Cell viability was measured 24 h later.

### 2.4 Crystal violet staining

Logarithmically growing HEI-OC1 were plated in a 6-well plate at a density of 15 × 10^4^ cells per well and incubated for 24 h at 37°C in a 5% CO_2_ atmosphere. Following a 2 h pretreatment with compound 19 at concentrations of 6.25, 12.5, and 50 μM, a cisplatin-induced injury model was established. The cells were then fixed with fixing solution for 30 min and stained with 0.1% crystal violet for 20 min, after which the cell number and morphology were observed.

### 2.5 Hoechst 33342 staining

HEI-OC1 cell death was assessed using Hoechst 33342 staining. Cells (5 × 10^4^/well) are cultured for 24 h, treated with compound 19 (6.25, 12.5, 25 μM) for 2 h, and exposed to cisplatin. After staining with Hoechst 33342 (10 mg/mL) in the dark for 15 min, the cells were examined under a fluorescence microscope (Thermo Fisher Scientific, China).

### 2.6 Live/dead cell analysis

HEI-OC1 cells (5 × 10⁴ cells/well) were seeded in 24-well plates. After treatment with the respective drugs for each group of cells, staining was performed with FDA (0.02 mg/mL) and PI (0.02 mg/mL) dyes and incubated in the dark for 10 min. The cells were then observed under a fluorescence microscope (Thermo Fisher Scientific, China) and photographed for analysis.

### 2.7 Flow cytometry detection

Each group of HEI-OC1 cells was treated and collected with trypsin, and all cells were resuspended in 500 μL of binding buffer according to the manufacturer’s instructions. PI dye and Annexin V dye were added and stained in the dark for 5/10 min. Cell analysis was carried out using a flow cytometer.

### 2.8 Transcriptome analysis

For RNA sequencing and transcriptome analysis, HEI-OC1 cells were seeded in 6-well plates at a density of 1 × 10^6^ cells per well. The experiment included cell-injury model and compound 19 groups (25 μM). Total RNA was extracted using TRIzol reagent. RNA purification, reverse transcription, library construction, and sequencing were performed by Shanghai Majorbio Biopharm Technology Co., Ltd. Gene abundance was measured by RSEM software. Differential expression analyses were performed using DESeq2 and DEGseq. Genes with |log2| ≥ 1 and FDR ≤ 0.05 (DESeq2) or FDR ≤ 0.001 (DEGseq) were considered as differentially expressed genes (DEGs). Kyoto Encyclopedia of Genes and Genomes (KEGG) pathway and gene ontology (GO) enrichment analyses of the DEGs were performed using Metascape and bioinformatics tools.

### 2.9 Quantitative reverse transcription polymerase chain reaction (RT-qPCR) analysis

Total RNA was extracted from cells of various groups using TRIzol reagent. The HiScript II Reverse Transcription Kit (Vazyme, China) was used for both reverse transcription and polymerase chain reaction (PCR) to measure expression levels. The primer sequences are listed in Supplementary Table S1.

### 2.10 Western blot analysis of related proteins

Total protein was extracted from HEI-OC1 cells and mouse cochlear tissues using RIPA buffer. Proteins were separated using 10% sodium dodecyl-sulfate polyacrylamide gel electrophoresis and transferred onto polyvinylidene difluoride membranes. After blocking with 5% skim milk, the membranes were rinsed with Tris buffered saline with Tween 20 and incubated with primary antibodies overnight. After further rinsing, the membranes were incubated with horseradish peroxidase-conjugated secondary antibodies for 2 h, rinsed again, and protein bands were visualized using an enhanced chemiluminescence reagent and imaged. Band densities were quantified using ImageJ software.

### 2.11 Immunofluorescence staining

Cells were fixed with 4% paraformaldehyde for 15 min, permeabilized with 0.2% Triton X-100 for 10 min, and then blocked with 5% BSA for 2 h. The primary antibody LC3B was incubated overnight at 4°C. Then, the FITC-labeled secondary antibody was incubated at 37°C in the dark for 30 min, followed by staining the nucleus with DAPI for 5 min. Finally, the cells were observed using a fluorescence microscope (Thermo Fisher Scientific, China).

### 2.12 Analysis of ros content

According to the instructions of the reactive oxygen species detection kit, cells were incubated with 2,7-dichlorofluorescein diacetate (DCFH-DA) for 30 min at 37°C in the dark. Then, cells were incubated with DAPI for 15 min to label the nucleus. The fluorescence intensity was observed using a fluorescence microscope (Thermo Fisher Scientific, China).

### 2.13 Detection of mitochondrial membrane potential

Prepare JC-1 buffer according to the method in the mitochondrial membrane potential (JC-1) kit instructions. Gently wash the cell surface, add JC-1 buffer, and incubate in the dark at 37°C for 30 min. After incubation, discard the liquid and observe and capture images under a fluorescence microscope (Thermo Fisher Scientific, China).

### 2.14 Molecular docking

The three-dimensional structure of compound 19 was drawn using Chem3D and energy-minimized before being saved as a Mol2 file. The AutoDock tool 1.5.6 was used to export the QPBQT file. The receptor protein FGFR1 (PDB ID: 5AM6) was downloaded from the Protein Data Bank. The docking center was located using Discovery Studio Visualizer 2020 software, and the water molecules and original ligands were removed. The processed receptor and ligand were imported into the AutoDock tool 1.5.6 and converted into PDBQT files. Molecular docking was performed using AutoDock, and the results were visualized using Discovery Studio Visualizer 2020.

### 2.15 Cell transfection

HEI-OC1 cells were transiently transfected using Lipofectamine 2000 (11,668,030, Invitrogen, Wuhan, China), strictly following the manual steps, seed the cells into a 6-well plate, and wait until the cell density is about 70 % for transfection. Preparation of transfection reagent: Solution A: Lipofectamine 2000 solution diluted in serum-free, antibiotic-free cell culture medium, mixed evenly; Solution B: The plasmid was diluted in serum-free, antibiotic-free cell culture medium and mixed uniformly. Solutions A and B were placed at room temperature for 5 min, then gently mixed in a 1:1 ratio and allowed to stand at room temperature for 10 min. Add 2 ml of regular cell culture medium containing serum and antibiotics to each well in the 6-well plate. Add the mixed transfection reagent to the transfection wells in the six-well plate, gently shake the culture plate to fully mix the transfection reagent with the cell culture medium, and culture in a cell incubator for 48 h, then proceed with subsequent operations. The sequences of ShLRP6 and NC are as follows: ShLRP6: CCG​ATG​CAA​TGG​AGA​TGC​AAA, NC: UUC​UCC​GAA​CGU​GUC​ACG​UTT.

### 2.16 Detection of related biochemical indicators

Using the oxidation kit purchased from Nanjing Jiancheng Bioengineering Company, strictly measure the level of lactate dehydrogenase (LDH) in the supernatant of HEI-OC1 cells according to the instructions. After lysing the cells, the oxidation kit is used to detect superoxide dismutase (SOD), malondialdehyde (MDA), and glutathione (GSH). In in vivo experiments, detecting the contents of SOD, MDA, and GSH in the cochlear tissue of mice and the level of LDH in mouse serum. Using a microplate reader (Multiskan FC, US), measure the optical density (OD) value at the corresponding wavelength and calculate the content of oxidative stress indicators according to the calculation formula.

### 2.17 Animals

Healthy male C57BL/6 mice (6 weeks old, weighing 22–25 g) were obtained from Changchun Yisi Laboratory Animal Technology Co., Ltd. They were placed in an environment with a 12-h light-dark cycle, and maintained at a constant humidity of 60% and a temperature of 25°C for adaptive feeding for 1 week to facilitate the mice to adapt to the environment. Strictly abiding by the “Animal Ethics Regulations of Jilin Agricultural University” and having obtained institutional approval (animal ethics approval number: 2023-KJT-021).

### 2.18 Induction of ototoxicity model and experimental design

Randomly, the mice required for the experiment were divided into 5 groups (8 mice/group), namely the blank control group, the cisplatin treatment group, the low-dose compound 19 group (10 mg/kg), the medium-dose compound 19 group (20 mg/kg), and the high-dose compound 19 group (40 mg/kg). Mice in the blank control group were administered intragastrically with 0.9% normal saline every day. Mice in the administration group were intragastrically administered compound 19. After the administration of compound 19 on the seventh day, except for the blank group, the other experimental groups were immediately intraperitoneally injected with cisplatin at a dose of 5 mg/kg for 5 days.

### 2.19 Detection of hearing threshold in mice by auditory brainstem response (ABR)

Mice were anesthetized with pentobarbital sodium (50 mg/kg, intraperitoneal injection). Recordings were made through three subcutaneous needle electrodes placed on the cranial vertex (active electrode), the right ear mastoid region (reference electrode), and the back (ground electrode). TDT System III (Tucker-Davis Technologies, United States) was used to generate stimuli and record responses, with 1,024 stimulus repetitions per recording. Acoustic stimuli were 10 ms tone bursts delivered through a broadband speaker (MF1; TDT) placed 10 cm in front of the animal’s head. ABR thresholds were tested at 8, 16, and 32 kHz. At each frequency, testing began at 90 dB SPL and tracked in 5 dB steps until the response disappeared.

### 2.20 Hair cell counting

The cochlear sections were permeabilized with 3% Triton x-100 for 45 min, then stained with DAPI staining solution for 10 min, and subsequently fixed with 50% glycerol. Observations and imaging were carried out using a fluorescence microscope. The number of hair cells was counted along the entire length of the cochlear epithelium from the apex to the base. The percentage of hair cell loss per 0.5 mm of epithelial cells and the length of the cochlea were plotted.

### 2.21 Histopathological analysis

Fix the dissected cochlea of mice in 4% paraformaldehyde solution for 72 h, perform section dewaxing and dehydration treatment, then embed it in paraffin, perform tissue sectioning, immerse the sections in hematoxylin staining solution for 2 min, and immediately wash in eosin solution for 1 min for staining. Observe under an optical microscope (Leica, DM750, Solms, Germany).

### 2.22 TUNEL staining

According to the instructions of the TUNEL kit (40307ES20, Yeasen Biotechnology, China), mix the sections with an appropriate amount of dUTP and buffer in the kit in a ratio of 1:5:50 and incubate in a 37°C incubator for 1 h. The nucleus was stained with DAPI staining solution for 15 min to locate the cells, sealed, and observed under a fluorescence microscope.

### 2.23 Transmission electron microscopy (TEM) analysis

Mouse cochlea tissues were immediately placed in 2.5% glutaraldehyde for fixation for electron microscopy. After repeated rinsing, the tissue was placed in 1% osmium tetroxide at 4°C for 2 h, then dehydrated with a gradient of acetone solutions (50%–90%) at room temperature. After dehydration, the tissue was embedded in pure acetone-EPON 812 embedding agent at room temperature. Ultra-thin sections of 1 μm and 50–70 nm were cut using an ultramicrotome, doubly stained with lead and uranium, and then dried. Under a transmission electron microscope, observations were made and photographic records were taken.

### 2.24 Statistical analysis

Data were statistically analyzed using GraphPad Prism 8.0, and the standard deviation was presented as the standard error of the mean. One-way analysis of variance was used for the statistical analysis of differences among three or more groups. Except for RNA sequencing analysis (Fisher’s exact test), statistical significance was determined using the Bonferroni post-hoc test. *p* < 0.05 was considered statistically significant.

## 3 Results

### 3.1 Establishment of cisplatin-induced ototoxicity model

**FIGURE 2:**
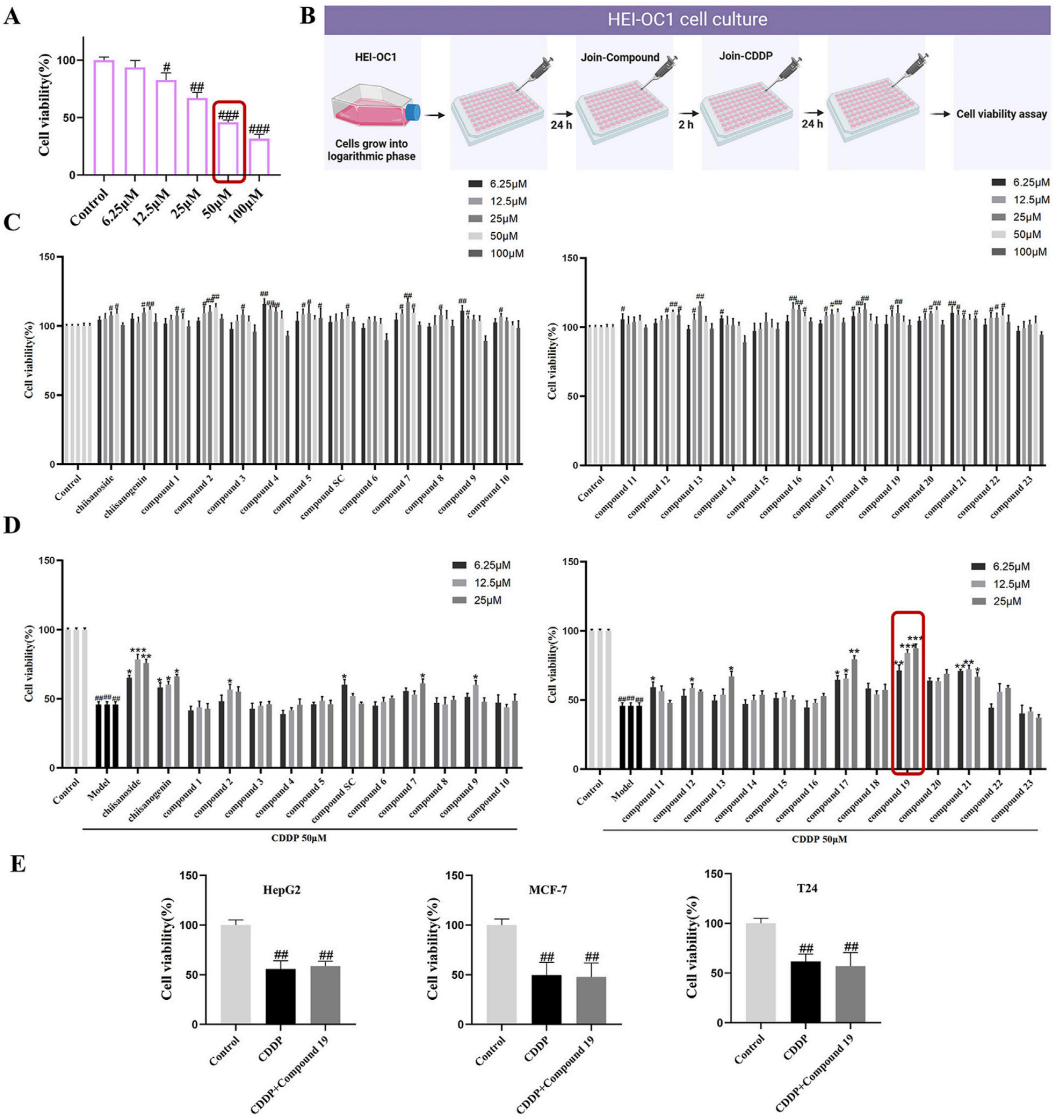
The protective activity of 26 candidate compounds against cisplatin-induced damage in HEI-OC1 cells. **(A)** The effect of varying concentrations of cisplatin on the activity of HEI-OC1. **(B)** In cell culture, compounds and cisplatin are administered in combination. HEI-OC1 cells are pretreated with various doses of compounds for 2 h before being exposed to cisplatin, and then treated with 50 μM cisplatin for 24 h. Created with Biorender.com. **(C)** Cytotoxicity assay of the compound on HEI-OC1 cells. **(D)** The protective activity of the compound against cisplatin-induced damage in HEI-OC1 cells.**(E)** The Impact of the Combined Administration of compound 19 and cisplatin on the Viability of HepG2, MCF-7 and T24 Cells.All data are presented as mean ± standard deviation (n = 3). Compared with the blank group, ^#^ *p* < 0.05, ^##^ *p* < 0.01,^###^ *p* < 0.005. Compared with the model group, *p < 0.05, **p < 0.01, ***p < 0.005.

To determine the appropriate dose of cisplatin, the CCK-8 method was used to detect in a gradient manner the effect of cisplatin at different concentrations (6.25–100 μM) on the viability of HEI-OC1 cells after treatment for 24 h. The results indicated that as the concentration of cisplatin increased, the activity of HEI-OC1 cells significantly decreased, presenting a dose-dependent trend. In subsequent experiments, 50 μM was selected as the modeling dose of cisplatin (*p* < 0.005; Figures 2A, B).

### 3.2 Protective effect of compounds on cisplatin-induced HEI-OC1 cell damage

Our early laboratory research discovered that chiisanoside, a distinctive secondary metabolite present in the leaves of *A. sessiliflorus*, exhibits protective effects against cisplatin-induced injury in HEI-OC1 cells. To enhance the clinical utility of this metabolite, we conducted a screening of the activity of chiisanoside-related derivatives synthesized in our laboratory to identify a candidate drug with superior activity for the prevention of cisplatin-induced ototoxicity.

In the preliminary experiment, after screening across a broad dose range, we found that excessive doses did not reflect significant drug effects; thus, concentration screening was limited to within 100 μM. The data revealed that the derivatives exhibited no significant cytotoxicity within the range of 0–25 μM (Figure 2C). Only a few compounds demonstrated inhibitory effects on cell proliferation at concentrations exceeding 50 μM. Therefore, for subsequent experiments, doses of 6.25, 12.5, and 25 μM were selected to investigate the cell-protective effects of the compounds on HEI-OC1 cells.

Active compounds at various concentrations were subjected to *in vitro* screening for activity in HEI-OC1 auditory hair cells. The results showed that chiisanogenin, compounds SC, 11, and 17 had protective effects on cisplatin-induced HEI-OC1 cell damage. The activity increased with the increase of the 31st position of the carbon chain and was the strongest when C = 3 and then decreased. The 28th carboxyl group of the lead compound chiisanogenin and compounds SC, 11, and 17 undergo acylation reaction with the amino group of organic amine. Among them, 2-ethoxyethylamine has the most significant protective activity on HEI-OC1 hair cells (Figure 2D, 87.54% ± 1.8%; *p* < 0.005), which is significantly higher than that of the model group (45.89% ± 2.03%). Therefore, we have reason to believe that compound 19 has a significant effect in preventing ototoxicity, and its protective effect is significantly higher than that of chiisanoside. Three concentrations (6.25, 12.5, and 25 μM) are selected for further activity verification and mechanism research.

### 3.3 The effects of compound 19 in combination with cisplatin on the viability of different cancer cells

To determine whether compound 19 affects the chemotherapeutic efficacy of cisplatin, the cell viability of HepG2, MCF-7, and T24 cells treated with compound 19 (25 μM) in combination with cisplatin was assessed using the CCK-8 method and compared with the group treated with cisplatin alone. As shown in Figure 2E, the results indicated that the cell activity was significantly reduced in the cisplatin group. At the same time, there was no significant difference in cell activity when compound 19 was co-administered with cisplatin. The findings suggest that compound 19 inhibits cisplatin-induced ototoxicity without affecting the anticancer activity of cisplatin.

### 3.4 The effect of compound 19 on inhibiting cisplatin-induced HEI-OC1 cell damage

**FIGURE 3:**
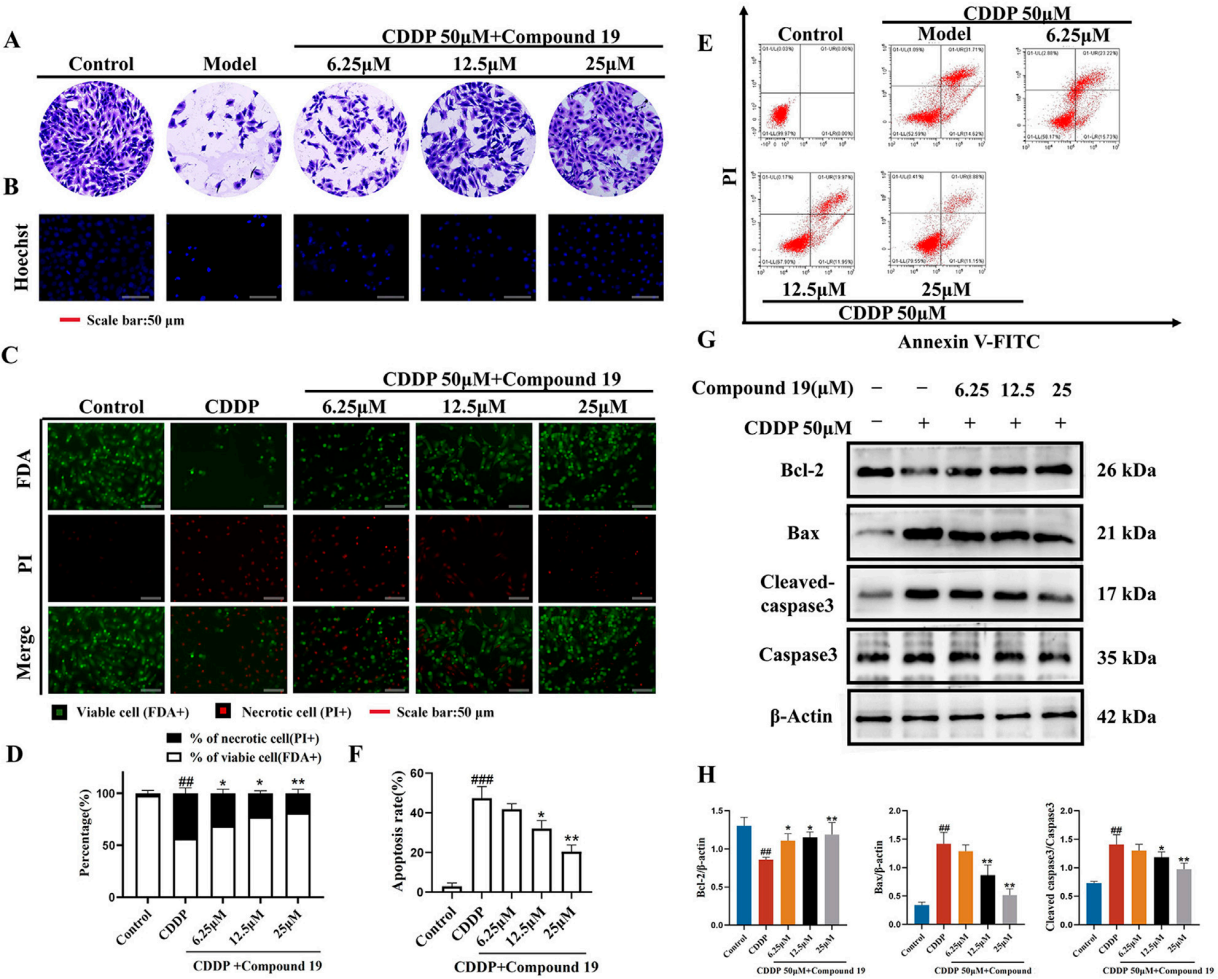
Protective effect of compound 19 on cisplatin-induced HEI-OC1 cell damage. **(A)** Morphological crystal violet staining of cells in each group. **(B)** Representative fluorescent micrographs of Hoechst 33342 staining of cells in each group. **(C)** Representative images of FDA (green)/PI (red) staining of HEI-OC1 cells. **(D)** Percentage of dead cells by FDA/PI staining. **(E)** Flow cytometry was used to analyze apoptosis and necrosis. Q1 represents necrotic cells (Annexin-V/PI −/+; upper left) and Q2 represents apoptotic cells (Annexin-V/PI +/+; upper right). **(F)** Calculate the percentages of viable cells, apoptotic cells and necrotic cells. **(G)** Detect the expression levels of Cleaved caspase-3, Caspase-3, Bax, and Bcl-2 proteins. **(H)** Quantify the relative protein content. All data are expressed as mean ± standard deviation (n = 3). Compared with the blank group, ^#^ *p* < 0.05, ^##^ *p* < 0.01, ^###^ *p* < 0.005. Compared with the model group, *p < 0.05, **p < 0.01.

Observing the crystal violet staining results of HEI-OC1 cells revealed that the number of live HEI-OC1 cells in the model group was significantly lower than that in the control group, and the cell morphology was significantly damaged (Figure 3A). In contrast, the viability of cells in the compound 19 pretreatment group was significantly increased, showing a dose-dependent trend and reaching a peak at 25 μM. Moreover, compared to the model group, the cells were arranged more neatly and had regular morphology. After Hoechst 33342 staining, it was found that the nuclei of HEI-OC1 cells in the control group were light blue, with uniform and regular shapes and in good condition (Figure 3B). After induction by cisplatin, nuclear division and distortion were observed, and the cells appeared bright blue. This was a morphological sign of apoptosis. However, pretreatment with different concentrations of compound 19 alleviated HEI-OC1 cell apoptosis.

Dual staining with fluorescein diacetate (FDA) and propidium iodide (PI) was used to determine the degree of cell damage induced by cisplatin (Figure 3C). Compared with the control group, a significant number of dead cells (red) were observed in the model group. Pretreatment with compound 19 significantly increased the uptake of FDA by living cells (green) and reduced the PI staining of dead cells (Figure 3D; *p* < 0.01). Flow cytometry analysis showed that compared with the control group, the percentage of apoptosis and necrosis of HEI-OC1 cells increased significantly after cisplatin treatment (Figures 3E, F; *p* < 0.005). Compared with the model group, the apoptosis and necrosis rate was significantly reduced after pretreatment with compound 19 (*p* < 0.01). These results indicate that compound 19 can effectively ameliorate cisplatin-induced apoptosis and necrosis of HEI-OC1 cells and play a pre-protective role against CIO.

The expression levels of apoptosis-related proteins in cisplatin-induced HEI-OC1 cells under the action of compound 19 were analyzed by Western blot (Figures 3G, H). Compared with the control group, under cisplatin treatment, the expression level of Cleaved caspase-3/Caspase-3 and Bax was significantly increased (*p* < 0.01), and the expression level of Bcl-2 protein was significantly decreased (*p* < 0.01); while compound 19 can ameliorate apoptosis of HEI-OC1 cells caused by cisplatin (*p* < 0.01). In conclusion, the results show that compound 19 can significantly reduce cisplatin-induced cell damage in HEI-OC1 cells.

### 3.5 RNA sequence analysis

**FIGURE 4:**
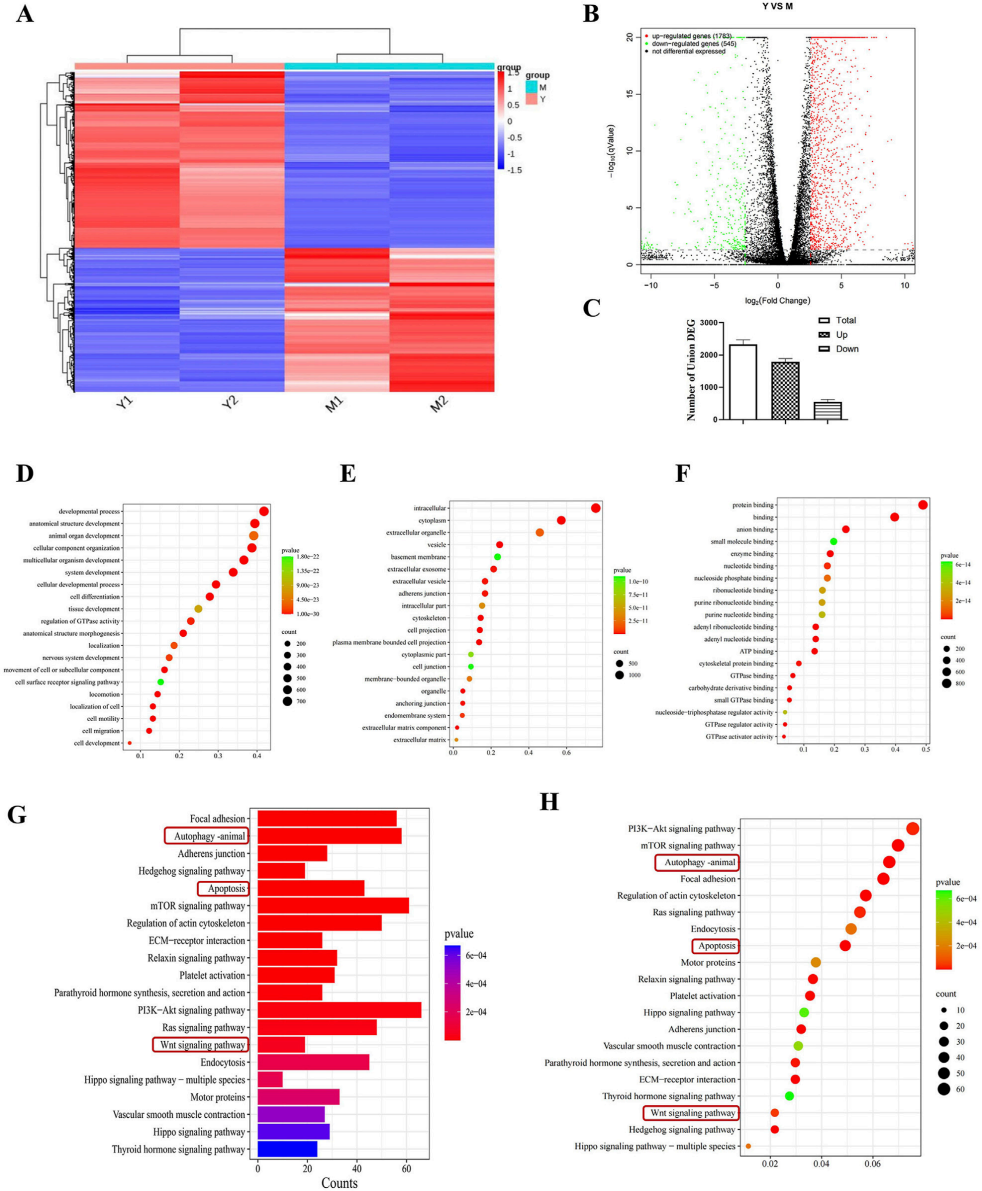
Transcriptomics analysis to screen for differential pathways. **(A)** Thermograms of all gene expression levels. In the figure, the abscissa represents the name of the differential gene and the ordinate represents the name of the sample table. Color represents the level of gene expression. **(B)** Volcanoes with all the different genes. **(C)** Histogram of total genes and differential genes (n = 2). **(D–F)** Show “biological process,” “cell component”and“molecular function” in GO analysis respectively. Each circle represents a GO term, and its expression distribution and significance are given. **(G, H)** Represents the histogram and bubble diagram of differential gene enrichment in KEGG pathway.

Transcriptome analysis was performed by selecting the CDDP group and the compound 19 (25 μM) group. Volcano plots and cluster analysis diagrams clearly present the differences in gene expression between the two groups of samples, and there is a high degree of repeatability. After comparing compound 19 with the control group, 2,328 differentially expressed genes (DEGs) were identified, among which 1783 genes were upregulated and 545 genes were downregulated (Figures 4A–C).

By means of Gene Ontology (GO) enrichment analysis, in-depth research on differentially expressed genes (DEGs) was conducted. We selected the top 20 functional categories for analysis, which cover biological processes, cellular components, and molecular functions. In biological processes (BP), DEGs are mainly involved in developmental processes, anatomical structure development, and animal organ development. In cellular components (CC), DEGs are mainly related to intracellular, cytoplasm, and extracellular organelle. In molecular functions (MF), DEGs are mainly associated with protein binding, binding, and anion binding (Figures 4D–F).

Through Kyoto Encyclopedia of Genes and Genomes (KEGG) pathway enrichment analysis, differentially expressed genes (DEGs) were studied. We selected the top 20 pathways with the smallest P value, which have the most reliable enrichment significance (Figures 4G, H). These pathways mainly include autophagy, apoptosis, and Wnt signaling pathways. Based on these findings, we selected autophagy and Wnt signaling pathways with significant differences for subsequent mechanism research.

### 3.6 Compound 19 activates autophagy in HEI-OC1 cells to reduce apoptosis

**FIGURE 5:**
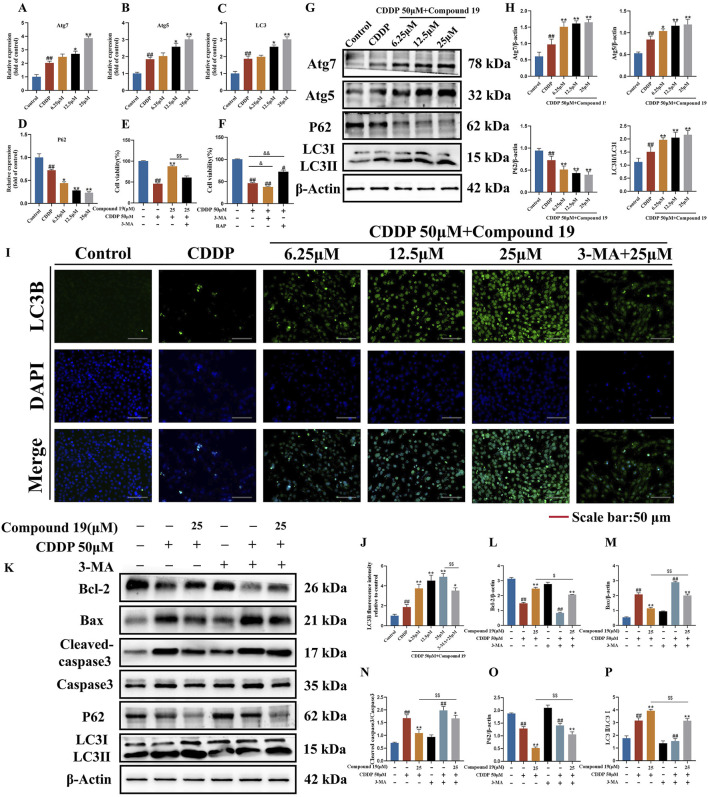
Effect of compound 19 on autophagy level after cisplatin exposure. **(A–D)** Detection of mRNA expression of autophagy-related differential genes Atg7, Atg5, P62 and LC3. **(E)** The viability of HEI-OC1 cells that are exposed to cisplatin and compound 19 with or without 3-MA. **(F)** Viability of HEI-OC1 cells after cisplatin exposure under treatment with 3-MA (5 mM) or rapamycin (0.1 μM). **(G, H)** Expression levels of Atg7, Atg5, P62 and LC3Ⅱ/Ⅰ proteins. **(I)** Immunofluorescence staining of LC3B in HEI-OC1 cells. **(J)** Relative fluorescence intensity of LC3B in HEI-OC1 cells. **(K)** Protein expression of Cleaved caspase-3, Caspase-3, Bax, Bcl-2, P62 and LC3II/I after treatment with 3-MA. **(L–P)** Quantitative analysis of protein expression after 3-MA treatment. All data are expressed as mean ± standard deviation (n = 3). Compared with the blank group, ^#^ *p* < 0.05, ^##^ *p* < 0.01. Compared with the model group, *p < 0.05, **p < 0.01. Compared with the compound 19 (25 μM) group, ^$^ *p* < 0.05, ^$$^ *p* < 0.01.

Based on the differentially expressed gene data, we selected autophagy pathway-related genes LC3, P62, Atg5, and Atg7 for reverse transcription-quantitative polymerase chain reaction (RT-qPCR) verification. The results showed (Figures 5A–D) that compared with the cisplatin (CDDP) group, compound 19 significantly increased the mRNA expression of LC3, Atg5, and Atg7 in HEI-OC1 cells after cisplatin damage, and the mRNA expression of P62 was significantly reduced (*p* < 0.01). In general, the relative expression of differentially expressed genes is consistent with the RT-qPCR results, indicating that the sequencing results are reliable.

To determine the effect of autophagy on cisplatin-induced HEI-OC1 cell damage, we treated HEI-OC1 cells with the autophagy inducer rapamycin (RAP) and the autophagy inhibitor 3-methyladenine (3-MA). The results of the CCK-8 assay showed (Figures 5E,F) that rapamycin increased the viability of HEI-OC1 cells after cisplatin treatment (*p* < 0.05), while 3-MA further accelerated cisplatin-induced HEI-OC1 cell damage (*p* < 0.01). The viability of HEI-OC1 cells in the cisplatin plus compound 19 group was significantly increased, and this effect was reversed by co-treatment with 3-MA (*p* < 0.01). It is proved that cisplatin-induced autophagy is protective autophagy. Activating autophagy can resist apoptosis of HEI-OC1 cells.

As shown in Figures 5G, H, after cisplatin exposure, the level of LC3 protein in HEI-OC1 cells increased simultaneously, indicating that cisplatin activates autophagy. At the same time, with the increase of the intervention concentration of compound 19, the expressions of autophagy-related proteins Atg5, Atg7 and LC3II/I after cisplatin induction were significantly upregulated (*p* < 0.01), and the expression of P62 was reduced (*p* < 0.01), and the protective autophagy in HEI-OC1 cells was activated. The analysis of immunofluorescence results shows that the fluorescence intensity of LC3 protein in the compound 19 group is significantly higher than that in the CDDP group (Figures 5I, J; *p* < 0.01). This indicates that cisplatin can activate autophagy expression in HEI-OC1 cells, but the treatment of compound 19 can further enhance autophagy and thus play a cell protective role.

To examine the role of activated autophagy in the process of compound 19 alleviating cisplatin-induced HEI-OC1 cell damage, the autophagy level was evaluated by comparing the protein expressions of LC3II/I and P62 after treatment with 3-MA (Figures 5K–P). After treatment with 3-MA, the expression of LC3II/I in the compound 19 group was inhibited, and the expression of P62 protein was increased (*p* < 0.01). At the same time, after treatment with 3-MA, the effects of compound 19 on the expressions of autophagy and apoptotic-related proteins were eliminated, and the expression levels of cleaved caspase-3/Caspase-3 and Bax were upregulated (*p* < 0.01), and the expression of Bcl-2 was reduced (*p* < 0.01). In conclusion, compound 19 antagonizes cisplatin-induced apoptosis in an autophagy-dependent manner. Autophagy plays a necessary role in alleviating CIO to a large extent.

### 3.7 Compound 19 activates autophagy and then effectively inhibits oxidative stress to resist cisplatin-induced HEI-OC1 cell damage

**FIGURE 6:**
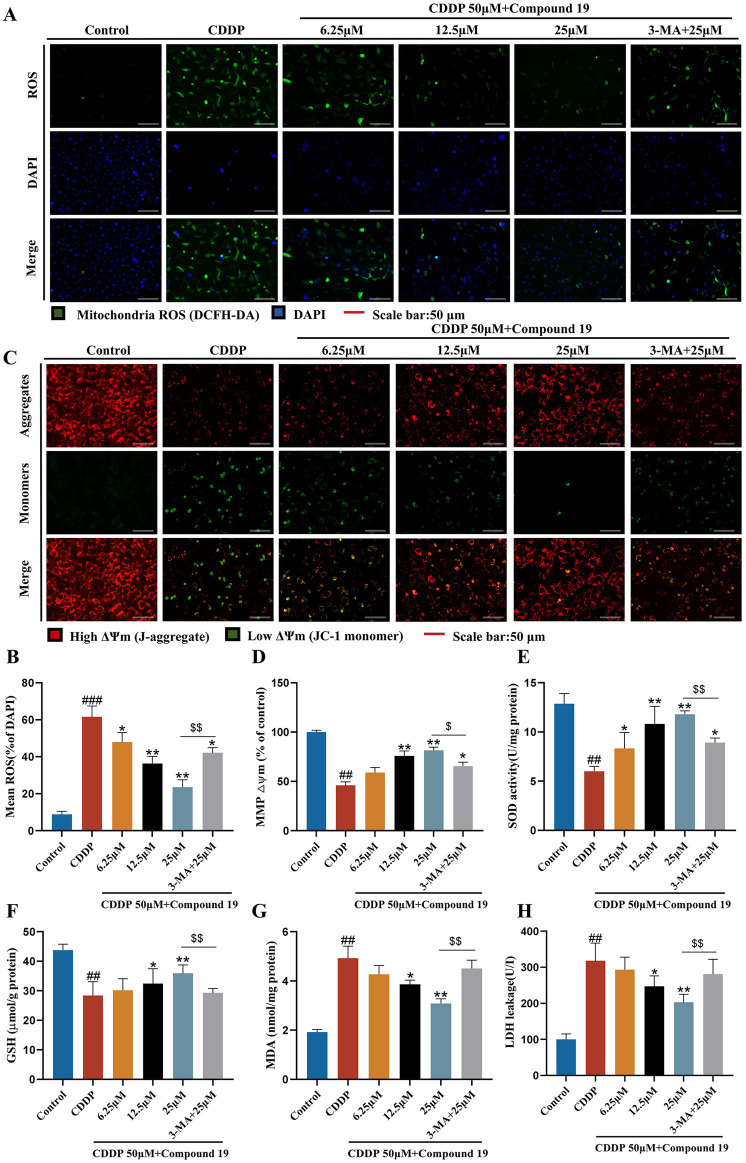
Protective effect of compound 19 on oxidative stress in cisplatin-induced HEI-OC1 cells. **(A, B)** Intracellular ROS was detected by DCFH-DA. ROS shows green fluorescence, and nuclei labeled by DAPI show blue fluorescence. **(C, D)** Mitochondrial membrane potential was detected by using JC-1 fluorescent probe. Generally, membrane potential shows red fluorescence. Decreased or lost mitochondrial membrane potential shows green fluorescence. **(E–H)** Levels of SOD, GSH, MDA and LDH in cells were detected. All data are expressed as mean ± standard deviation (n = 3). Compared with the blank group, ^#^ *p* < 0.05, ^##^ *p* < 0.01, ^###^ *p* < 0.005. Compared with the model group, *p < 0.05, **p < 0.01. Compared with the compound 19 (25 μM) group, ^$^ *p* < 0.05, ^$$^ *p* < 0.01.

It has been reported that excessive reactive oxygen species (ROS) is one of the potential mechanisms of cisplatin-induced ototoxicity (Callejo et al., 2015). Antioxidants are effective protectors against cisplatin-induced hearing loss (Kim et al., 2018).To investigate whether compound 19 protects cisplatin-induced apoptosis by activating autophagy and inhibiting oxidative stress, we used the DCFH-DA probe to evaluate the intracellular ROS level in HEI-OC1 cells (Figures 6A, B). The results showed that after cisplatin exposure, the ROS level in HEI-OC1 cells was significantly increased (*p* < 0.005), compound 19 significantly inhibited the accumulation of ROS (*p* < 0.01), and compared with the administration group, the oxidative stress level in the cisplatin + compound 19 + 3-MA group was significantly increased (*p* < 0.01), indicating that compound 19 reduces the oxidative stress level of damaged cells by up-regulating autophagy.

The decline of mitochondrial membrane potential is one of the early events of apoptosis. Studies have shown that the autophagy inhibitor 3-methyladenine can increase the levels of apoptosis-related protein Bax and cytochrome C by reducing the mitochondrial membrane potential of L-02 cells in the *in vitro* model of acute liver failure (ALF), thereby counteracting the anti-hepatocyte apoptosis effect mediated by autophagy (Chen et al., 2019). To determine whether compound 19 can prevent the decline of mitochondrial membrane potential through autophagy, we used the JC-1 fluorescent probe to detect changes in mitochondrial membrane potential (Figures 6C, D). After cisplatin damage, the mitochondrial membrane potential declines, but there is no obvious decline in the compound 19 group. The autophagy inhibitor prevents the protective effect of compound 19 on mitochondria (*p* < 0.01). This proves that compound 19 prevents the decline of mitochondrial membrane potential and apoptosis in cisplatin-induced HEI-OC1 cells by activating autophagy.

To explore the antioxidant efficacy of compound 19, the changes in the content of major antioxidant enzymes in cells were tested (Figures 6E–H). After cisplatin treatment, the contents of superoxide dismutase (SOD) and glutathione (GSH) in cells were significantly reduced (*p* < 0.01), while the content of malondialdehyde (MDA) and the exposure rate of lactate dehydrogenase (LDH) were significantly increased (*p* < 0.01). Compound 19 can alleviate the downward trend of SOD and GSH contents caused by cisplatin (*p* < 0.01), and with the increase of its dose, it can significantly reduce the MDA level and reduce LDH exposure (*p* < 0.01). After applying the autophagy inhibitor 3-MA, this treatment group significantly counteracted the antioxidant effect of compound 19 (*p* < 0.01). In conclusion, these results indicate that compound 19 can significantly reduce the oxidative damage of HEI-OC1 cells induced by cisplatin. Further proof shows that compound 19 exerts a protective effect by effectively inhibiting oxidative stress through activating autophagy.

### 3.8 Compound 19 protects cisplatin-induced HEI-OC1 cell damage through LRP6/GSK3β

**FIGURE 7:**
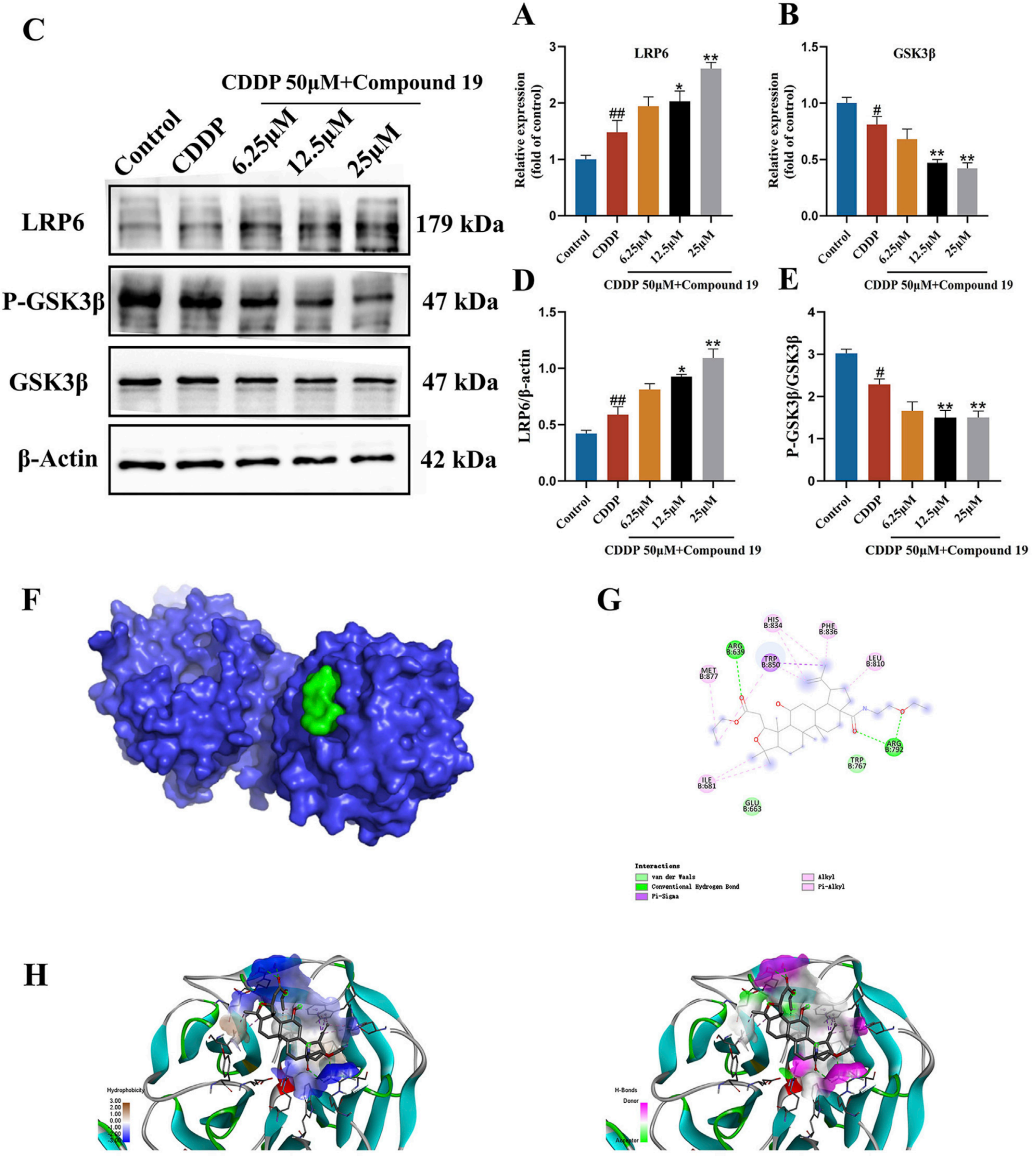
Effect of compound 19 on the LRP6/GSK3β pathway in HEI-OC1 cells. **(A, B)** Changes in mRNA expression levels of LRP6 and GSK3β. **(C)** Protein expression of LRP6, P-GSK3β and GSK3β. **(D, E)** Quantitative analysis of LRP6 and P-GSK3β/GSK3β proteins. **(F)** The degree of spatial tessellation between compound 19 and the LRP6 protein domain. **(G)** The planar binding conformation of compound 19 with the LRP6 protein (the interaction force with the amino acid residues of the protein). **(H)** The hydrogen bonding force between compound 19 and the LRP6 protein and the conformation of the hydrophobic and hydrophilic fields. All data are expressed as mean ± standard deviation (n = 3). Compared with the blank group, ^#^ *p* < 0.05, ^##^ *p* < 0.01. Compared with the model group, *p < 0.05, **p < 0.01.

Pathway function enrichment analysis was performed on potential targets in transcriptome data to determine the molecular pathway involved in the autophagy-promoting effect of compound 19. Among all potential pathways, since GSK3β is an important regulator of autophagy and LRP6 has been shown to attenuate GSK3β activity, based on the previous differentially expressed gene (DEG) data, we observed an upregulation of the LRP6 gene and a downregulation of the GSK3β gene (Supplementary Table S3). Therefore, it was hypothesized that compound 19 triggers the autophagy process in HEI - OC1 cells by inhibiting GSK3β via LRP6. To validate this hypothesis, we examined each group’s mRNA and protein levels of LRP6 and GSK3β. The results are shown in Figures 7A–E. It was found that although cisplatin slightly increased the mRNA and protein levels of LRP6, these levels were significantly upregulated after treatment with compound 19 (*p* < 0.01). Compared with the model group, the gene expression levels of GSK3β were significantly reduced dose-dependent (*p* < 0.01). The expressions of GSK3β protein were detected by Western blotting. Compared with the model group, p-GSK3β/GSK3β was significantly reduced under compound 19 treatment (*p* < 0.01). In conclusion, compound 19 may activate autophagy to resist cisplatin-induced apoptosis by activating LRP6, thereby inhibiting GSK3β.

### 3.9 Simulated binding effect of compound 19 and LRP6 protein

We performed molecular docking on LRP6 treated with compound 19. The docking score is-5.7 kcal/mol, which is less than-5 kcal/mol (Figure 7F). Compound 19 is tightly embedded in the active pocket of the LRP6 protein. The interaction mode between LRP6 and compound 19 is as follows: compound 19 interacts with residues ARG 792 and ARG 639 by using conventional hydrogen bonds, forms Pi-Sigma type force with residue TRP850, interacts with residues TRP 767 and GLU 633 through van der Waals force, and has hydrophobic interactions with other residues MET 877, ILE 681, HIS 834, PHE 836 and LEU 810 (Figures 7G, H). This shows that compound 19 has an appropriate molecular weight and spatial distribution and can be well bound to the active pocket.

### 3.10 The LRP6/GSK3β pathway is the key pathway through which compound 19 activates autophagy in HEI-OC1 cells and inhibits cisplatin-induced apoptosis

**FIGURE 8:**
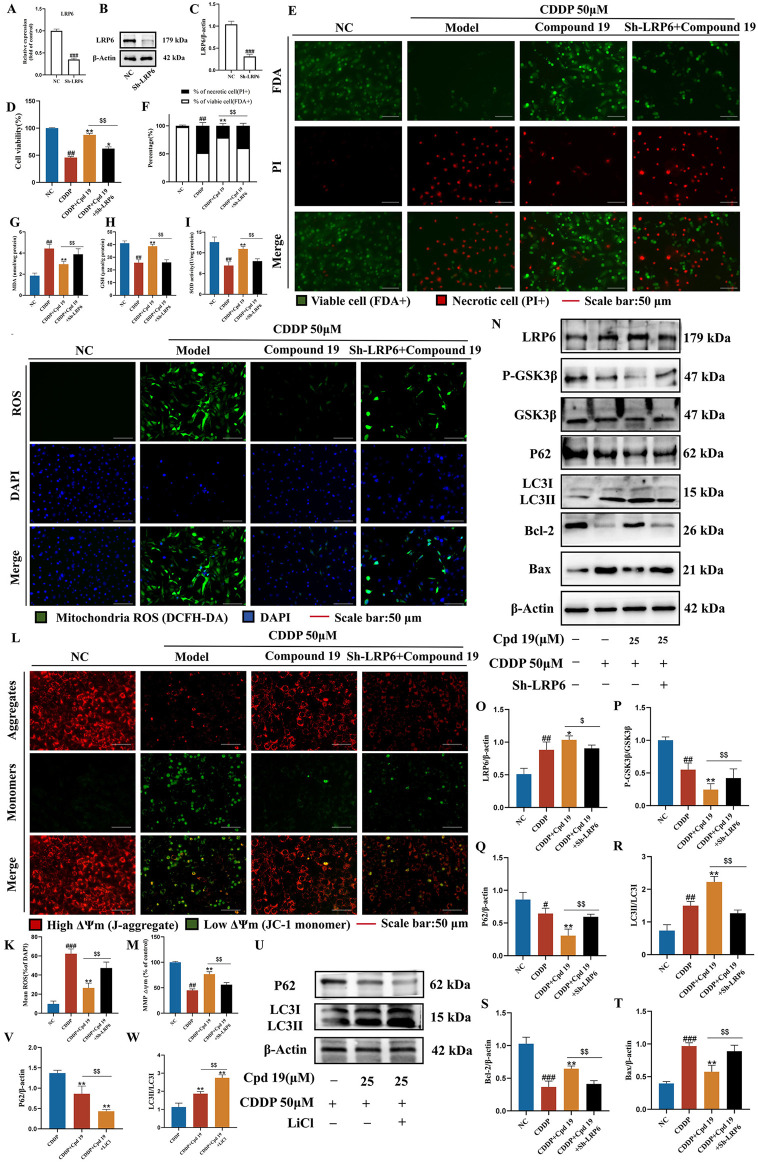
LRP6 is a key target for compound 19 to activate autophagy and inhibit apoptosis. **(A–C)** Detection of gene and protein expression levels after LRP6 gene knockdown. **(D)** Effect of down-regulating LRP6 on cell viability. **(E, F)** FDA/PI staining level of HEI-OC1 cells after LRP6 knockdown. **(G–I)** Detection of intracellular MDA, GSH and SOD levels after LRP6 knockdown. **(J, K)** Detection of intracellular ROS level after LRP6 knockdown. **(L, M)** Detection of intracellular mitochondrial membrane potential level after LRP6 knockdown. **(N–T)** Detection of protein expression levels of LRP6, P-GSK3β/GSK3β, P62, LC3Ⅱ/Ⅰ, Bcl-2 and Bax. **(U–W)** Detect the protein expression levels of P62 and LC3Ⅱ/Ⅰ after LiCl treatment. All data are expressed as mean ± standard deviation (n = 3). Compared with the blank group, ^#^ *p* < 0.05, ^##^ *p* < 0.01, ^###^ *p* < 0.005. Compared with the model group, *p < 0.05, **p < 0.01. Compared with the compound 19 (25 μM) group, ^$^ *p* < 0.05, ^$$^ *p* < 0.01.

To determine whether the protection of compound 19 against cisplatin-induced cell damage by activating autophagy is indeed caused by up-regulating LRP6, we investigated whether inhibiting LRP6 expression can reverse the autophagy level and reverse the protective effect of compound 19 on HEI-OC1 cells. We knocked down LRP6 using sh-LRP6 plasmids and confirmed the reduction of LRP6 protein and gene expression in HEI-OC1 cells. (Figures 8A–C). The results of CCK-8 and FDA/PI showed that knockdown of LRP6 reversed the pre-protective effect of compound 19 on cells and significantly promoted cell death (Figures 8D–F; *p* < 0.01).

Compared with the compound 19 group, after knocking down LRP6, the level of MDA, a typical lipid peroxidation product, significantly increased in cells (Figure 8G; *p* < 0.01), while the levels of GSH and SOD decreased (Figures 8H, I; *p* < 0.01). Next, the levels of intracellular ROS and mitochondrial membrane potential were detected by DCFH-DA and JC-1 respectively. The results showed that compared with the compound 19 group, after knocking down LRP6, the ROS level significantly increased (Figures 8J, K; *p* < 0.01), and the mitochondrial membrane potential significantly decreased and approached that of the CDDP group (Figures 8L, M; *p* < 0.01). In general, these data indicate that knocking down LRP6 can promote the accumulation of lipid peroxides and the production of ROS, reverse the protective effect of compound 19 on HEI-OC1 cells, and thus promote cisplatin-induced apoptosis.

Western blot results demonstrated that, compared with the compound 19 group, the relevant proteins were reversed after LRP6 was knocked down. The ratio of p-GSK3β/GSK3β was significantly decreased under the treatment of compound 19, while the deficiency of LRP6 in HEI-OC1 cells led to the opposite pattern. (Figures 8N–P; *p* < 0.01). After knocking down LRP6 in the presence of compound 19, the levels of key autophagy proteins LC3Ⅱ/Ⅰ and P62 were reversed, indicating a significant reduction in autophagy level (Figures 8N, Q, R; *p* < 0.01). In addition, by detecting the level of apoptotic proteins, it was found that the effect of compound 19 was eliminated after knockdown, and the protein expression was close to that of the CDDP group. Knocking down LRP6 promoted apoptosis (Figures 8N, S, T; *p* < 0.01).

Then, we utilized LiCl to inhibit GSK3β and examined its influence on autophagy. The results demonstrated that the inhibition of GSK3β augmented autophagy. This was manifested by the increased level of LC3 II/I and the decreased level of P62 in the presence of LiCl compared with the treatment with compound 19 alone (Figures 8U–W; *p* < 0.01). Furthermore, GSK3β may enhance autophagy and lysosomal biogenesis by affecting the nuclear translocation of TFEB (Supplementary Figure S3; *p* < 0.01). In summary, these results suggest that compound 19 activates autophagy by stimulating the LRP6/GSK3β signaling pathway following cisplatin damage, thereby protecting cells from cisplatin-induced injury.

### 3.11 Study on the inhibitory effect of compound 19 on cisplatin-induced ototoxicity *in vivo*

**FIGURE 9:**
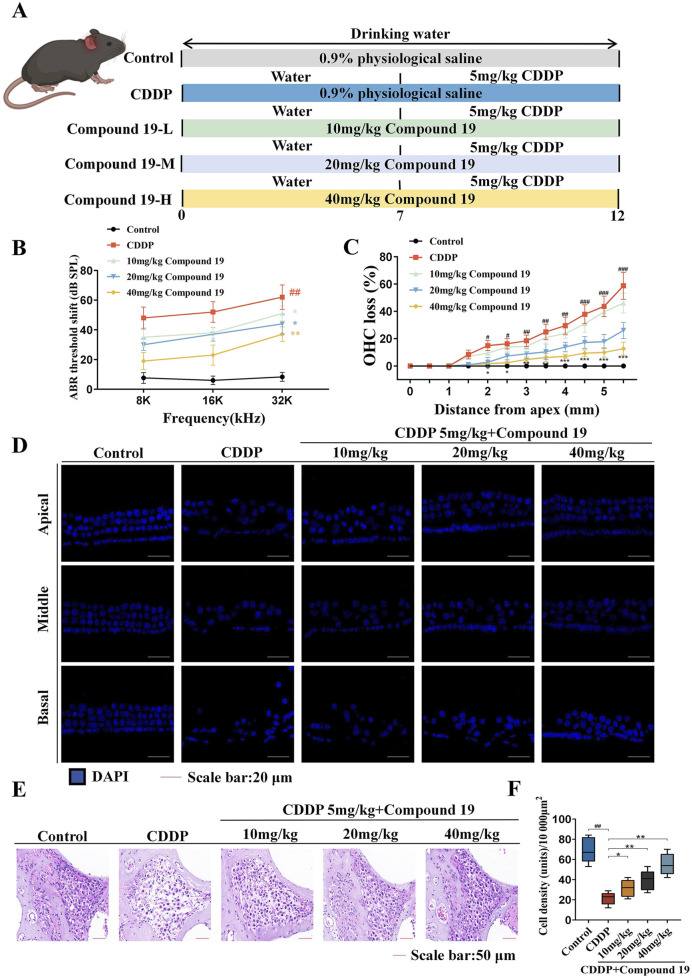
Protective effect of compound 19 on cisplatin-induced ototoxicity in C57BL/6 mice. **(A)** Experimental protocol for compound 19 in treating cisplatin-induced ototoxicity in mice. **(B)** ABR thresholds of mice in each group. **(C)** Percentage of outer hair cell loss calculated from five independent cochlear dissections from the apical, middle and basal turns. **(D)** DAPI staining of representative cochlear sections from the apical, middle and basal turns. **(E)** Spiral ganglion observed by H&E staining. **(F)** Quantitative analysis of spiral ganglion density. All data are expressed as mean ± standard deviation (n = 8). Compared with the blank group, ^#^ *p* < 0.05, ^##^ *p* < 0.01, ^###^ *p* < 0.005. Compared with the model group, *p < 0.05, **p < 0.01, ***p < 0.005.

Next, we explored the protective potential of compound 19 on cisplatin-induced hearing loss *in vivo*. C57BL/6 mice were orally administered with different doses of compound 19 every day. After administration on the seventh day, cisplatin (5 mg/kg, 5 days) was injected intraperitoneally to induce hearing loss in mice and establish a cisplatin injury model (Figure 9A). An auditory brainstem response (ABR) test was performed 24 h after cisplatin injection on the last day. During the experiment, the body weight of mice in each group was recorded (Supplementary Figure S4).

The ABR test was used to evaluate the hearing level of mice in each group (Figure 9B). The results showed that compared with the control group, the ABR threshold of mice in the cisplatin pretreatment group increased at all frequencies, confirming that cisplatin exposure leads to severe hearing loss (*p* < 0.01). Compound 19 significantly reduced the auditory threshold shift at 8, 16 and 32 kHz mediated by CDDP (*p* < 0.01), proving that compound 19 effectively deals with cisplatin-induced hearing loss *in vivo*.

Platinum-based drugs mainly damage outer hair cells (OHCs) and then damage spiral ganglion neurons (SGNs) (Tang et al., 2021b). Based on the effective protective effect of compound 19 on mouse hearing, we explored whether compound 19 can rescue hair cell damage caused by cisplatin. The damage of each component of hair cells was examined by DAPI staining. As shown in Figures 9C, D, in the control group, one row of inner hair cells (IHCs) and three rows of outer hair cells (OHCs) were neatly arranged, and there was no loss from the apex to the basal turn. Cisplatin treatment led to cell loss and disorder of cochlear hair cells. The missing outer hair cells were mainly located at the basal turn (*p* < 0.005). In addition, the morphology and arrangement of hair cells under the pre-protection of compound 19 were significantly better than those in the CDDP group. Compound 19 significantly reduced the loss of OHCs after CDDP exposure (*p* < 0.01). These results indicate that compound 19 can normalize morphology and reduce the loss of hair cells.

Since the spiral ganglion (SGN) is also regarded as an important target for judging the hearing level, we marked the cells contained in the SGN area in the middle part of the mouse cochlea by H&E staining (Figures 9E, F). Compared with the control group, the number of spiral ganglion cells per unit area in the CDDP group decreased, with a large number of cells lost and a large number of vacuole-like changes. The cell nuclei were deformed, dissolved, and even disappeared (*p* < 0.01). Compared with the CDDP group, the morphology of cochlear cells in the compound 19 group with a concentration of 10 mg/kg did not change significantly. After pre-protection with compound 19 at concentrations of 20 mg/kg and 40 mg/kg, the number of spiral ganglion cells increased, the cytoplasm became full, the cell nuclei gradually became larger, and the vacuole-like degeneration gradually decreased (*p* < 0.01). The results show that compound 19 has an effective protective effect on cisplatin-induced hair cell damage and spiral ganglion loss, and has rescued the hearing loss induced by CDDP. This further confirms that compound 19 is a promising ear protectant.

### 3.12 The inhibitory effect of compound 19 on cisplatin-induced apoptosis *in vivo*

**FIGURE 10:**
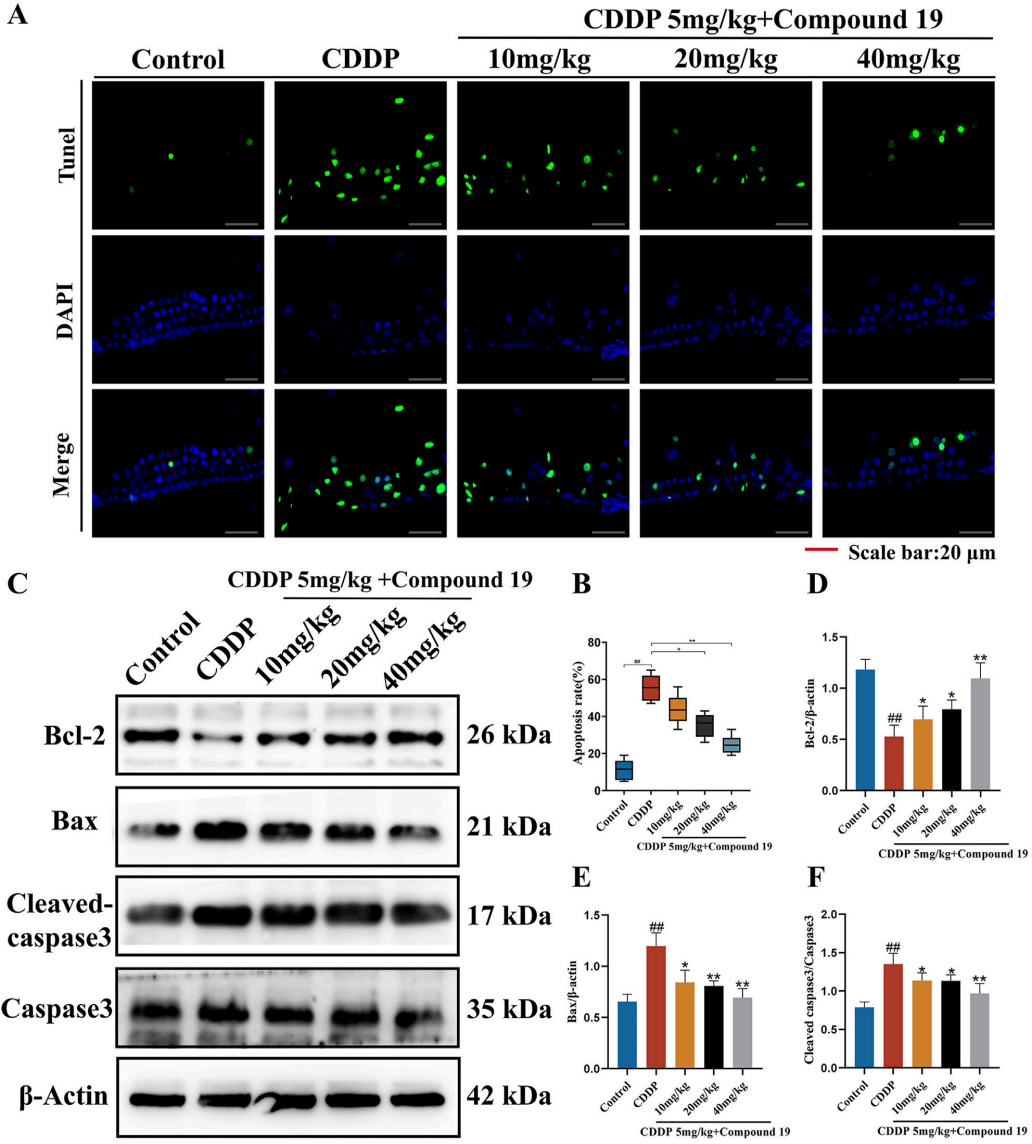
The inhibitory effect of compound 19 on cisplatin-induced apoptosis *in vivo*. **(A, B)** Detection of apoptosis by TUNEL staining. **(C)** Expression levels of Bcl-2, Bax, cleaved caspase-3 and Caspase-3 proteins in the cochlea. **(D–F)** Quantitative analysis of proteins in the cochlea. All data are expressed as mean ± standard deviation (n = 8). Compared with the blank group, ^#^ *p* < 0.05, ^##^ *p* < 0.01. Compared with the model group, *p < 0.05, **p < 0.01.

To prove the important role of compound 19 in cisplatin-induced apoptosis of hair cells, TUNEL staining was used to observe and quantify the TUNEL positive rate of outer hair cells in each group. As shown in Figures 10A, B, apoptotic cells were marked with green fluorescence, and the nuclei were marked with DAPI blue fluorescence. Compared with the Control group, the apoptosis rate of outer hair cells in the CDDP group increased, and there was a significant difference compared with the Control group (*p* < 0.01). After pretreatment of hair cells with compound 19, only a small number of TUNEL-positive cells were observed (*p* < 0.01). The results showed that cisplatin induced apoptosis, resulting in the death of cochlear hair cells and supporting cells and presenting ototoxicity. The pre-protection of compound 19 could significantly inhibit cisplatin-induced apoptosis.

The results of the Western blot experiment (Figures 10C–F) showed that cisplatin significantly increased the expressions of the pro-apoptotic genes Bax and Cleaved caspase 3/Caspase 3 and significantly decreased the expression of the anti-apoptotic gene Bcl-2 (*p* < 0.01). After the addition of compound 19, the expression of Bcl-2 was significantly increased, and the expressions of Bax and Cleaved caspase 3/Caspase 3 were significantly decreased (*p* < 0.01). The above results indicate that compound 19 plays an important role in cisplatin-induced apoptosis of hair cells and can protect hair cells from cisplatin-induced apoptosis.

### 3.13 The influence of compound 19 on autophagy activation and related proteins

**FIGURE 11:**
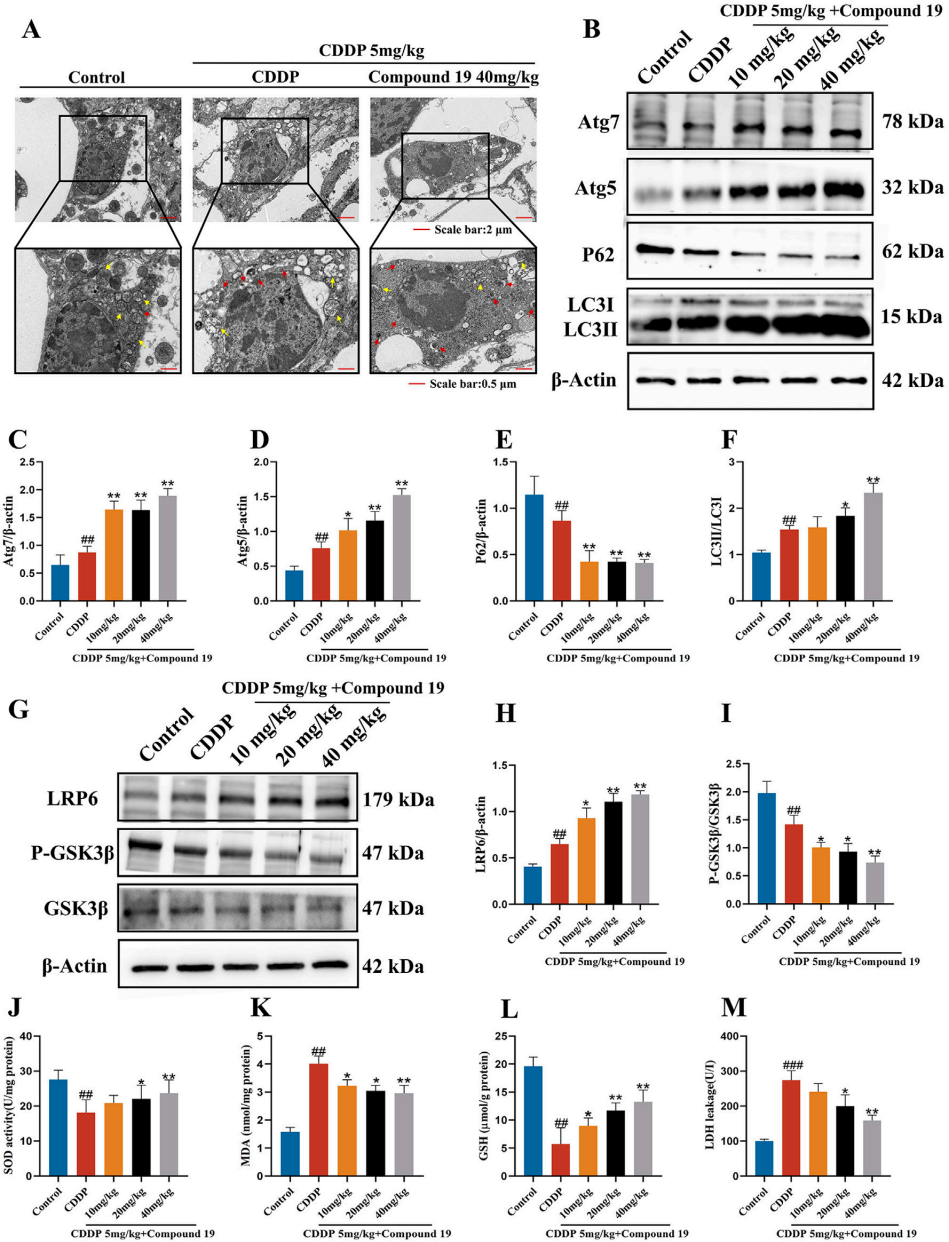
Compound 19 protects CIO by activating autophagy and then inhibiting oxidative stress through the LRP6/GSK3β axis. **(A)** TEM images of mouse OHC. The yellow arrows represent mitochondria, and the red arrows represent autophagosomes. **(B–F)** Expression levels of autophagy-related proteins Atg7, Atg5, P62 and LC3Ⅱ/Ⅰ in the cochlea. **(G–I)** Detection of protein expression levels of LRP6, P-GSK3β and GSK3β in the cochlea. **(J–M)** Detection of SOD, GSH, MDA levels in the cochlea and LDH level in the serum. All data are expressed as mean ± standard deviation (n = 8). Compared with the blank group, ^#^ *p* < 0.05, ^##^ *p* < 0.01. Compared with the model group, *p < 0.05, **p < 0.01.

Transmission electron microscope (TEM) images show that disordered structures and loss of cristae in mitochondria are observed in the cisplatin-treated group (yellow arrows). The number of autophagosomes (red arrows) in the compound 19 group is higher than that in the CDDP group, indicating that compound 19 has promoted the activation of autophagosomes (Figure 11A; *p* < 0.01). This suggests that compound 19 has activated the autophagy expression level in cochlear hair cells, and the protective effect of compound 19 on CIO may depend on the activation of autophagy.

Similarly, we analyzed the expression levels of autophagy marker proteins by Western blotting to study the expression of autophagy in cochlear hair cells caused by cisplatin under the influence of compound 19. As shown in Figures 11B–F, in the cisplatin-treated group, the contents of LC3II/I, Atg5, and Atg7 increased significantly, while the content of P62 decreased (*p* < 0.01). After pre-protection with compound 19, with the increase of the intervention concentration of compound 19, the expressions of Atg5, Atg7, and LC3II/I increased significantly, and the expression of P62 decreased, indicating that autophagy was activated (*p* < 0.01). This is consistent with the results of cell experiments. Autophagy may be one of the potential mechanisms to alleviate cisplatin-induced ototoxicity.

Based on the results of cell experiments, we verified the protein expression of upstream kinases of autophagy, including LRP6 and GSK3β, through *in vivo* experiments. It was found that compound 19 activates LRP6 and inhibits GSK3β activity during cisplatin induction in cochlear hair cells, thereby promoting autophagy and exerting a protective effect (Figures 11G–I; *p* < 0.01). It further verified that compound 19 activates autophagy, inhibits hair cell apoptosis, and thus protects mice from CIO damage by stimulating the LRP6/GSK3β signaling pathway.

### 3.14 Compound 19 enhances antioxidant capacity *in vivo*

To study the effect of compound 19 on the biochemical indices of oxidative stress in mice induced by cisplatin, we measured the activity of malondialdehyde (MDA), the main marker of lipid peroxidation, and the antioxidant enzymes superoxide dismutase (SOD), glutathione (GSH) and lactate dehydrogenase (LDH). The results are shown in Figures 11J–M. Compared with the control group, the MDA level in the cochlear tissue of mice treated with cisplatin increased significantly (*p* < 0.01), and this increase was effectively reversed after treatment with compound 19 (*p* < 0.01). In the CDDP group, the activities of SOD and GSH in the cochlear tissue decreased significantly (*p* < 0.01), and the activities of key antioxidant enzymes were significantly inhibited. Treatment with compound 19 effectively improved this situation (*p* < 0.01). In addition, the decrease in GSH level further supported the argument that the oxidative stress state was enhanced (*p* < 0.01). After cisplatin exposure, the LDH index in the serum of mice increased significantly, and it decreased in a dose-dependent manner after treatment with compound 19 (*p* < 0.01). Therefore, compound 19 enhances the antioxidant capacity *in vivo*, which is consistent with the results of *in vitro* experiments. Therefore, compound 19 enhances the antioxidant capacity *in vivo*. Consistent with the results of *in vitro* experiments, compound 19 activates autophagy through the LRP6/GSK3β pathway, inhibits the generation of oxidative stress in HEI-OC1 cells and mouse cochlear hair cells induced by cisplatin, thereby preventing apoptosis and inhibiting CIO damage.

## 4 Discussion and conclusion

Cisplatin has become one of the most widely used chemotherapeutic agents for various solid tumors in clinical practice due to its high efficacy and broad spectrum (Qi et al., 2019). However, its potential to cause irreversible ototoxicity has severely limited its clinical application. Cisplatin is the cis-isomer of [PtCl_4_(NH_3_)_2_] and exists in the form of a dichloro complex, and then permeates into the inner ear through the stria vascularis (Huang et al., 1995). After cisplatin enters the outer hair cells, CTR2 promotes the accumulation of platinum within the cells (Sun et al., 2016). With the assistance of copper transporters (CTR1 and CTR2) and the organic cation transporter-2 (OCT2), cisplatin crosses the apical membrane of the hair cells. Under the condition of low intracellular chloride ion concentration (4 mM), cisplatin undergoes a hydration reaction and is converted into its active state (Omar and Elewa, 2023; Wang et al., 2023). Cisplatin interacts with nucleotides and amino acids, triggering DNA cross-linking and protein misfolding in the cellular environment (Huang et al., 1995; Omar and Elewa, 2023). The platinum-DNA adduct formed after cisplatin enters the nucleus activates several signaling pathways, leading to cell death (Huang et al., 1995; Lim et al., 2024).

Based on the fact that chiisanoside, the main component in the leaves of *A. sessiliflorus*, has good activity in resisting cisplatin-induced HEI-OC1 cell damage, this study carried out activity screening on 26 seco-lupane-type triterpene derivatives related to chiisanoside and investigated the mechanism of their ototoxicity protection.The results of cell activity screening showed that compound 19 has a significant protective effect on cisplatin-induced damage to HEI-OC1 cells, and its protective effect is significantly higher than that of chiisanoside. By analyzing the effects of compound 19 on tumor cells, it was found that compound 19 can inhibit cisplatin-induced damage to auditory hair cells without affecting the anticancer activity of cisplatin. Experiments such as crystal violet staining and Western blot showed that compound 19 significantly reversed the cisplatin-induced cell morphological distortion, damage and irregular arrangement, and inhibited cell apoptosis and necrosis, showing excellent anti-ototoxicity effects. In order to clarify the mechanism of action of compound 19, transcriptome sequencing analysis was carried out on the optimal dose components of HEI-OC1 cells pre-protected by compound 19. GO and KEGG pathway enrichment analysis showed that autophagy, apoptosis and Wnt signaling pathways were the most significant signaling pathways related to CIO damage.

Autophagy is a crucial process involved in the development and functional maturation of the cochlea (Hayashi et al., 2015; Yuan et al., 2015; Fujimoto et al., 2017). LC3 and P62 are important markers in autophagy. It has been confirmed that LC3, as an autophagy indicator, participates in the synthesis of autophagosomes through the conversion of soluble LC3-I to LC3-II, representing the occurrence of autophagy (Barth et al., 2010). The activation of autophagy leads to the degradation of the autophagy substrate P62. The accumulation of P62 is usually related to the inhibition of autophagy, so its level can be used as an indication of the autophagy state (Pankiv et al., 2007). ATG5, a key protein involved in the extension of the phagocytic membrane to autophagic vesicles, plays an important role in autophagy (Changotra et al., 2022). ATG7 is an E-1 enzyme that participates in the activation of ubiquitin-like proteins (UBL), such as ATG12 and ATG8, and transfers them to the E-2 enzyme. It is an indispensable key factor in the autophagy process and is crucial for the formation of autophagosomes in the classical pathway (Zhu et al., 2019). Apoptosis is a form of actively regulated cell death, which is mainly involved in CIO (Ruhl et al., 2019). Autophagy is an important part of apoptosis, packaging damaged cell components before cell lysis. Autophagy can also play a role in the survival of cells under stress conditions (such as starvation and oxidative stress) by preventing the spread of intracellular and extracellular damage (Rabinowitz and White, 2010). Experiments have confirmed that after pretreatment with compound 19, the protein and mRNA expression levels of ATG5, ATG7, and LC3II/I increase, while the expression level of P62 decreases. As expected, by detecting the levels of related apoptotic proteins, it was found that treatment with the autophagy inhibitor (3-MA) reversed the protective effect of compound 19 on ear hair cells by inhibiting autophagy. Therefore, compound 19 resists cisplatin-induced apoptosis by activating autophagy.

It has been reported that when the accumulation of free radicals exceeds the scavenging capacity of the antioxidant defense system, peroxidative damage will occur in tissue cells (Görlach et al., 2015). Oxidative stress can cause oxidative damage to DNA and abnormal protein expression, resulting in toxic effects on cells (Sies, 2015). Many studies have also demonstrated that oxidative stress caused by reactive oxygen species is a key link in apoptosis (Chandra et al., 2000; Kannan and Jain, 2000). Excessive reactive oxygen species (ROS) are the main factors causing auditory cell damage by cisplatin (Jeong et al., 2007; Callejo et al., 2015). Excessive ROS can damage or consume the antioxidant defense system, affect the function of antioxidant enzymes such as SOD, and promote the generation of lipid peroxides such as 4-HNE. There is a close interaction between ROS and autophagy: ROS can act as a cell signaling molecule to trigger autophagy (Scherz-Shouval et al., 2007b; Chen et al., 2008), while autophagy can reduce oxidative damage by engulfing and degrading oxidized substances (Scherz-Shouval et al., 2007a; He et al., 2017). In this study, compound 19 can activate autophagy, inhibit oxidative stress, and prevent the decline of mitochondrial membrane potential at the same time. The addition of the autophagy inhibitor (3-MA) significantly reversed the above-mentioned protective effects. This further indicates that compound 19 can inhibit the production of oxidative stress in HEI-OC1 and mouse cochlear hair cells induced by cisplatin by activating the autophagy pathway, thereby preventing cell apoptosis.

The LRP6/GSK3β signaling axis is a major autophagy monitoring system among the differentially expressed genes in transcriptomics. LRP6 (Low-density lipoprotein receptor-related protein 6) is a transmembrane protein that functions in cell signaling, especially playing an important role in the Wnt/β-catenin signaling pathway. LRP6 inhibits the activity of GSK3β (Wang et al., 2020). Studies have demonstrated that enhancing autophagy by inhibiting GSK3β can alleviate cisplatin-induced ototoxicity (Liu et al., 2019). GSK3β can affect autophagy expression through related pathways. For example, after inhibiting GSK3β, it promotes the nuclear translocation of TFEB, thereby increasing autophagy (Theeuwes et al., 2020). Experiments have confirmed that compound 19 significantly upregulates the protein and mRNA expression of LRP6 in HEI-OC1 cells and mouse cochlear hair cells, leading to a decrease in the expression of p-GSK3β/GSK3β.

To verify that LRP6 serves as the main target for inhibiting GSK3β to activate cellular autophagy and resist cisplatin-induced apoptosis, we knocked down the LRP6 protein in HEI-OC1 cells. The results showed that the knockdown of LRP6 reversed the pre-protective effect of compound 19 on the cells. The expression of p-GSK3β/GSK3β was significantly increased, the levels of autophagy-related proteins P62 and LC3II/I were reversed, autophagy was inhibited, the levels of ROS and MDA were elevated, while the levels of GSH and SOD were decreased. The mitochondrial membrane potential was close to that of the CDDP group, and the apoptosis level of HEI-OC1 cells was significantly increased. As mentioned previously, our results indicated that LRP6 regulates the activity of GSK3β. After treating the cells with the GSK3β inhibitor LiCl, the level of autophagy was enhanced. This further clarified that compound 19 activates autophagy through the LRP6/GSK3β pathway to inhibit oxidative stress and then suppress cisplatin-induced ototoxicity.

In the current study, the ABR assay was used to show that compound 19 protects mice from hearing loss after cisplatin exposure. By studying the morphological changes of cochlear hair cells (HCs) after treatment with compound 19, it was further confirmed that compound 19 could protect the inner ear auditory function from damage caused by cisplatin. The damage of the spiral ganglion (SGN) is a precursor to hearing loss (Duan et al., 2024). Compound 19 can also protect against cisplatin-induced SGN damage, which is one of the reasons why compound 19 has significant activity in resisting cisplatin-induced ototoxicity and hearing loss in mice. The molecular docking results show that compound 19 is tightly embedded in the active pocket of the LRP6 protein. Compound 19 has excellent binding properties due to its appropriate molecular weight and spatial distribution.

**FIGURE 12:**
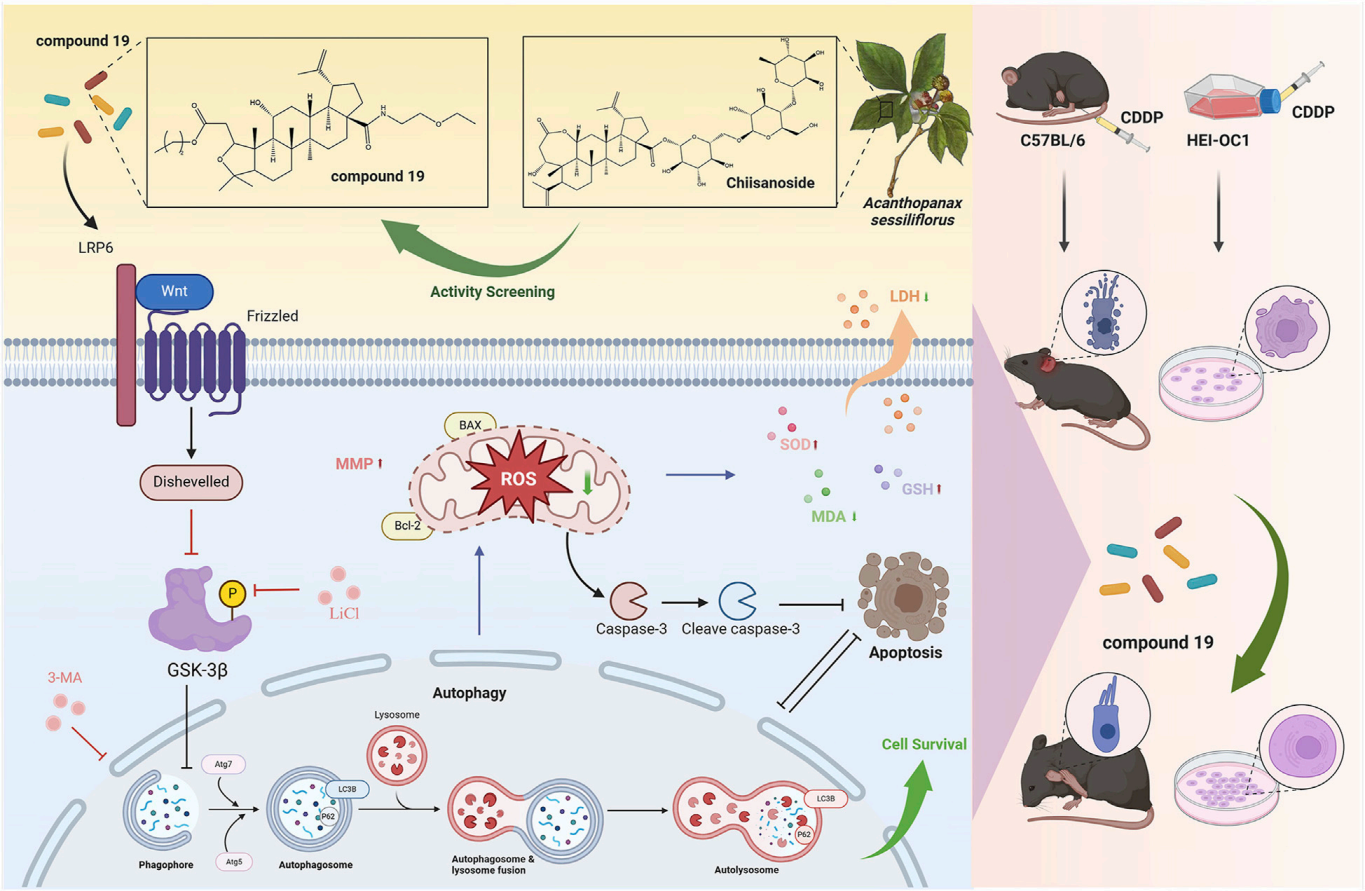
The compound 19, a derivative of *Acanthopanax sessiliflorus* leaves, activates autophagy through the LRP6/GSK3β axis, thereby inhibiting oxidative stress and exerting an anti-cisplatin-induced ototoxicity effect. The figure was created with Biorender.com.

In summary, this study found that compound 19, a chiisanoside derivative from the leaves of *A. sessiliflorus*, has a significant effect in resisting cisplatin-induced ototoxicity in mice. This protective effect mainly activates autophagy through the LRP6/GSK3β axis, thereby inhibiting oxidative stress and apoptosis, and at the same time protecting cochlear hair cells and spiral ganglions from damage and loss (Figure 12). It is an innovative discovery that compound 19 can be used as an autophagy activator with excellent activity to counteract cisplatin-induced hair cell damage. It is found that compound 19 can be used as an autophagy activator with excellent activity to resist cisplatin-induced hair cell damage, and LRP6 can be used as a new target for the treatment and prevention of CIO, which provides a new way for the treatment of sensorineural deafness. In conclusion, compound 19 can be used as an autophagy activator with strong development potential to prevent cisplatin-induced ototoxicity.

## Data Availability

The original contributions presented in the study are included in the article/Supplementary Material, further inquiries can be directed to the corresponding authors.

## Abbreviations

Acanthopanax sessiliflorus, A. sessiliflorus; HEI-OC1, Mouse cochlear hair cell lines; CDDP, Cisplatin; CIO, Cisplatin-induced ototoxicity; Cpd 19, compound 19; DMEM, Reagents Dulbecco’s Modified Eagle’s Medium; CCK-8, Cell Counting Kit-8; PBS, Phosphate-buffered saline; 3-MA, 3-methyladenine; RAP, Rapamycin; RNAi, RNA interference; BCA, bicinchoninic acid; BP, biological process; CC, cellular component; MF, molecular function; DEGs, differentially expressed genes; FBS, fetal bovine serum; GO, gene ontology; KEGG, Kyoto Encyclopedia of Genes and Genomes; TBST, Tris-buffered saline with Tween-20; ABR, Auditory brainstem response; OHC, outer hair cell; IHC, inner hair cells; SGN, Spiral Ganglion Neurons; H&E, hematoxylin-eosin staining; MMP, mitochondrial membrane potential; TEM, transmission electron microscope; ROS, reactive oxygen species.

## References

[B1] Adachi M. Yanagizono K. Okano Y. Koizumi H. Uemaetomari I. Tabuchi K. (2023). Estradiol protects hair cells from cisplatin-induced ototoxicity via Nrf2 activation. Redox Rep. 28 (1), 2161224. 10.1080/13510002.2022.2161224 36661237 PMC9869986

[B2] Anfuso C. D. Cosentino A. Agafonova A. Zappalà A. Giurdanella G. Trovato Salinaro A. (2022). Pericytes of stria vascularis are targets of cisplatin-induced ototoxicity: new insights into the molecular mechanisms involved in blood-labyrinth barrier breakdown. Int. J. Mol. Sci. 23 (24), 15790. 10.3390/ijms232415790 36555432 PMC9781621

[B3] Barth S. Glick D. Macleod K. F. (2010). Autophagy: assays and artifacts. J. Pathol. 221 (2), 117–124. 10.1002/path.2694 20225337 PMC2989884

[B4] Bian X. Liu X. Liu J. Zhao Y. Li H. Zhang L. (2019). Hepatoprotective effect of chiisanoside from Acanthopanax sessiliflorus against LPS/D-GalN-induced acute liver injury by inhibiting NF-κB and activating Nrf2/HO-1 signaling pathways. J. Sci. Food Agric. 99 (7), 3283–3290. 10.1002/jsfa.9541 30552777

[B5] Callejo A. Sedó-Cabezón L. Juan I. D. Llorens J. (2015). Cisplatin-induced ototoxicity: effects, mechanisms and protection strategies. Toxics 3 (3), 268–293. 10.3390/toxics3030268 29051464 PMC5606684

[B6] Chandra J. Samali A. Orrenius S. (2000). Triggering and modulation of apoptosis by oxidative stress. Free Radic. Biol. Med. 29 (3-4), 323–333. 10.1016/s0891-5849(00)00302-6 11035261

[B7] Changotra H. Kaur S. Yadav S. S. Gupta G. L. Parkash J. Duseja A. (2022). ATG5: a central autophagy regulator implicated in various human diseases. Cell Biochem. Funct. 40 (7), 650–667. 10.1002/cbf.3740 36062813

[B8] Chen L. Xin X. Feng H. Li S. Cao Q. Wang X. (2020). Isolation and identification of anthocyanin component in the fruits of Acanthopanax sessiliflorus (rupr. and Maxim.) seem. By means of high speed counter current chromatography and evaluation of its antioxidant activity. Molecules 25 (8), 1781. 10.3390/molecules25081781 32295006 PMC7221754

[B9] Chen Q. Wang Y. Jiao F. Z. Shi C. X. Gong Z. J. (2019). Histone deacetylase 6 inhibitor ACY1215 offers a protective effect through the autophagy pathway in acute liver failure. Life Sci. 238, 116976. 10.1016/j.lfs.2019.116976 31634464

[B10] Chen Y. McMillan-Ward E. Kong J. Israels S. J. Gibson S. B. (2008). Oxidative stress induces autophagic cell death independent of apoptosis in transformed and cancer cells. Cell Death Differ. 15 (1), 171–182. 10.1038/sj.cdd.4402233 17917680

[B11] Duan B. Peng K. A. Wang L. (2024). Injury and protection of spiral ganglion neurons. Chin. Med. J. 137 (6), 651–656. 10.1097/cm9.0000000000002765 37407223 PMC10950135

[B12] Fujimoto C. Iwasaki S. Urata S. Morishita H. Sakamaki Y. Fujioka M. (2017). Autophagy is essential for hearing in mice. Cell Death and Dis. 8 (5), e2780. 10.1038/cddis.2017.194 PMC5520715 28492547

[B13] Gao X. Mao H. Zhao L. Li X. Liao Y. Li W. (2024). Nuciferine protects cochlear hair cells from ferroptosis through inhibiting NCOA4-mediated ferritinophagy. Antioxidants 13 (6), 714. 10.3390/antiox13060714 38929153 PMC11201048

[B14] Görlach A. Dimova E. Y. Petry A. Martínez-Ruiz A. Hernansanz-Agustín P. Rolo A. P. (2015). Reactive oxygen species, nutrition, hypoxia and diseases: problems solved? Redox Biol. 6, 372–385. 10.1016/j.redox.2015.08.016 26339717 PMC4565025

[B15] Guo L. Cao W. Niu Y. He S. Chai R. Yang J. (2021). Autophagy regulates the survival of hair cells and spiral ganglion neurons in cases of noise, ototoxic drug, and age-induced sensorineural hearing loss. Front. Cell. Neurosci. 15, 760422. 10.3389/fncel.2021.760422 34720884 PMC8548757

[B16] Hayashi K. Dan K. Goto F. Tshuchihashi N. Nomura Y. Fujioka M. (2015). The autophagy pathway maintained signaling crosstalk with the Keap1–Nrf2 system through p62 in auditory cells under oxidative stress. Cell. Signal. 27 (2), 382–393. 10.1016/j.cellsig.2014.11.024 25435427

[B17] He Z. Guo L. Shu Y. Fang Q. Zhou H. Liu Y. (2017). Autophagy protects auditory hair cells against neomycin-induced damage. Autophagy 13 (11), 1884–1904. 10.1080/15548627.2017.1359449 28968134 PMC5788479

[B18] Huang H. Zhu L. Reid B. R. Drobny G. P. Hopkins P. B. (1995). Solution structure of a cisplatin-induced DNA interstrand cross-link. Science 270 (5243), 1842–1845. 10.1126/science.270.5243.1842 8525382

[B19] Jeong H.-J. Kim S.-J. Moon P.-D. Kim N.-H. Kim J.-S. Park R.-K. (2007). Antiapoptotic mechanism of cannabinoid receptor 2 agonist on cisplatin-induced apoptosis in the HEI-OC1 auditory cell line. J. Neurosci. Res. 85 (4), 896–905. 10.1002/jnr.21168 17183590

[B20] Kannan K. Jain S. K. (2000). Oxidative stress and apoptosis. Pathophysiology 7 (3), 153–163. 10.1016/s0928-4680(00)00053-5 10996508

[B21] Kim D. Heo Y. Kim M. Suminda G. G. D. Manzoor U. Min Y. (2024a). Inhibitory effects of Acanthopanax sessiliflorus Harms extract on the etiology of rheumatoid arthritis in a collagen-induced arthritis mouse model. Arthritis Res. and Ther. 26 (1), 11. 10.1186/s13075-023-03241-1 38167214 PMC10763440

[B22] Kim G.-D. Lee J. Auh J.-H. (2024b). Metabolomic screening of anti-inflammatory compounds in Acanthopanax sessiliflorus fruit (Ogaza) extract. Appl. Biol. Chem. 67 (1), 56. 10.1186/s13765-024-00912-8

[B23] Kim K.-H. Lee B. Kim Y.-R. Kim M.-A. Ryu N. Jung D. J. (2018). Evaluating protective and therapeutic effects of alpha-lipoic acid on cisplatin-induced ototoxicity. Cell Death and Dis. 9 (8), 827. 10.1038/s41419-018-0888-z PMC6070527 30068942

[B24] Kroemer G. Mariño G. Levine B. (2010). Autophagy and the integrated stress response. Mol. Cell 40 (2), 280–293. 10.1016/j.molcel.2010.09.023 20965422 PMC3127250

[B25] Lee D.-Y. Seo K.-H. Lee D.-S. Kim Y.-C. Chung I.-S. Kim G.-W. (2012). Bioactive 3,4-seco-Triterpenoids from the fruits of Acanthopanax sessiliflorus. J. Nat. Prod. 75 (6), 1138–1144. 10.1021/np3002173 22691179

[B26] Lee J.-W. Baek N.-I. Lee D.-Y. (2015). Inhibitory effects of seco-triterpenoids from Acanthopanax sessiliflorus fruits on HUVEC invasion and ACE activity. Nat. Product. Commun. 10 (9), 1517–1520. 10.1177/1934578X1501000907 26594747

[B27] Li H. Song Y. He Z. Chen X. Wu X. Li X. (2018). Meclofenamic acid reduces reactive oxygen species accumulation and apoptosis, inhibits excessive autophagy, and protects hair cell-like HEI-OC1 cells from cisplatin-induced damage. Front. Cell Neurosci. 12, 139. 10.3389/fncel.2018.00139 29875633 PMC5974247

[B28] Lim K. H. Park S. Han E. Yoon H. S. Lee Y. Hong S. (2024). Protective effects of Y-27632 against cisplatin-induced ototoxicity: a zebrafish model Y-27632 and cisplatin-induced ototoxicity. Food Chem. Toxicol. 190, 114792. 10.1016/j.fct.2024.114792 38849049

[B29] Liu T. Zong S. Luo P. Qu Y. Wen Y. Du P. (2019). Enhancing autophagy by down-regulating GSK-3β alleviates cisplatin-induced ototoxicity *in vivo* and *in vitro* . Toxicol. Lett. 313, 11–18. 10.1016/j.toxlet.2019.05.025 31220555

[B30] Marullo R. Werner E. Degtyareva N. Moore B. Altavilla G. Ramalingam S. S. (2013). Cisplatin induces a mitochondrial-ROS response that contributes to cytotoxicity depending on mitochondrial redox status and bioenergetic functions. PLoS One 8 (11), e81162. 10.1371/journal.pone.0081162 24260552 PMC3834214

[B31] Mi K. Dolan P. J. Johnson G. V. W. (2006). The low density lipoprotein receptor-related protein 6 interacts with glycogen synthase kinase 3 and attenuates activity. J. Biol. Chem. 281 (8), 4787–4794. 10.1074/jbc.M508657200 16365045

[B32] Omar N. E. Elewa H. (2023). Cisplatin-induced ototoxicity: a novel approach to an ancient problem. Pharmacogenetics Genomics 33 (5), 111–115. 10.1097/fpc.0000000000000497 37068004

[B33] Pan H. Y. Valapala M. (2022). Regulation of autophagy by the glycogen synthase kinase-3 (GSK-3) signaling pathway. Int. J. Mol. Sci. 23 (3), 1709. 10.3390/ijms23031709 35163631 PMC8836041

[B34] Pankiv S. Clausen T. H. Lamark T. Brech A. Bruun J. A. Outzen H. (2007). p62/SQSTM1 binds directly to Atg8/LC3 to facilitate degradation of ubiquitinated protein aggregates by autophagy. J. Biol. Chem. 282 (33), 24131–24145. 10.1074/jbc.M702824200 17580304

[B35] Park H.-J. Kim H.-J. Bae G.-S. Seo S.-W. Kim D.-Y. Jung W.-S. (2009). Selective GSK-3beta inhibitors attenuate the cisplatin-induced cytotoxicity of auditory cells. Hear. Res. 257 (1), 53–62. 10.1016/j.heares.2009.08.001 19666099

[B36] Prasad K. Borre E. D. Dillard L. K. Ayer A. Der C. Bainbridge K. E. (2024). Priorities for hearing loss prevention and estimates of global cause-specific burdens of hearing loss: a systematic rapid review. Lancet Glob. Health 12 (2), e217–e225. 10.1016/S2214-109X(23)00514-4 38245112

[B37] Qi L. Luo Q. Zhang Y. Jia F. Zhao Y. Wang F. (2019). Advances in toxicological research of the anticancer drug cisplatin. Chem. Res. Toxicol. 32 (8), 1469–1486. 10.1021/acs.chemrestox.9b00204 31353895

[B38] Rabinowitz J. D. White E. (2010). Autophagy and metabolism. Science 330 (6009), 1344–1348. 10.1126/science.1193497 21127245 PMC3010857

[B39] Ruhl D. Du T.-T. Wagner E. L. Choi J. H. Li S. Reed R. (2019). Necroptosis and apoptosis contribute to cisplatin and aminoglycoside ototoxicity. J. Neurosci. 39 (15), 2951–2964. 10.1523/jneurosci.1384-18.2019 30733218 PMC6462451

[B40] Scherz-Shouval R. Shvets E. Elazar Z. (2007a). Oxidation as a post-translational modification that regulates autophagy. Autophagy 3 (4), 371–373. 10.4161/auto.4214 17438362

[B41] Scherz-Shouval R. Shvets E. Fass E. Shorer H. Gil L. Elazar Z. (2007b). Reactive oxygen species are essential for autophagy and specifically regulate the activity of Atg4. Embo J. 26 (7), 1749–1760. 10.1038/sj.emboj.7601623 17347651 PMC1847657

[B42] Sies H. (2015). Oxidative stress: a concept in redox biology and medicine. Redox Biol. 4, 180–183. 10.1016/j.redox.2015.01.002 25588755 PMC4309861

[B43] Sun S. Cai J. Yang Q. Zhao S. Wang Z. (2016). The association between copper transporters and the prognosis of cancer patients undergoing chemotherapy: a meta-analysis of literature and datasets. Oncotarget 8 (9), 16036–16051. 10.18632/oncotarget.13917 PMC5362544 27980217

[B44] Tang Q. Wang X. Jin H. Mi Y. Liu L. Dong M. (2021a). Cisplatin-induced ototoxicity: updates on molecular mechanisms and otoprotective strategies. Eur. J. Pharm. Biopharm. 163, 60–71. 10.1016/j.ejpb.2021.03.008 33775853

[B45] Tang Q. Wang X. Jin H. Mi Y. Liu L. Dong M. (2021b). Cisplatin-induced ototoxicity: updates on molecular mechanisms and otoprotective strategies. Eur. J. Pharm. Biopharm. 163, 60–71. 10.1016/j.ejpb.2021.03.008 33775853

[B46] Teng H. Wu D. Lu L. Gao C. Wang H. Zhao Y. (2023). Design and synthesis of 3,4-seco-lupane triterpene derivatives to resist myocardial ischemia-reperfusion injury by inhibiting oxidative stress-mediated mitochondrial dysfunction via the PI3K/AKT/HIF-1α axis. Biomed. and Pharmacother. 167, 115452. 10.1016/j.biopha.2023.115452 37688986

[B47] Theeuwes W. F. Gosker H. R. Schols A. M. W. J. Langen R. C. J. Remels A. H. V. (2020). Regulation of PGC-1α expression by a GSK-3β-TFEB signaling axis in skeletal muscle. Biochimica Biophysica Acta (BBA) - Mol. Cell Res. 1867 (2), 118610. 10.1016/j.bbamcr.2019.118610 31738957

[B48] Wang H. Yu W. Zhang D. Zhao Y. Chen C. Zhu H. (2022). Cytotoxic and anti-tumor effects of 3,4-seco-lupane triterpenoids from the leaves of Eleutherococcus sessiliflorus against hepatocellular carcinoma. Nat. Prod. Res. 36 (4), 1062–1066. 10.1080/14786419.2020.1844698 33183092

[B49] Wang X. Zhou Y. Wang D. Wang Y. Zhou Z. Ma X. (2023). Cisplatin-induced ototoxicity: from signaling network to therapeutic targets. Biomed. and Pharmacother. 157, 114045. 10.1016/j.biopha.2022.114045 36455457

[B50] Wang Y. Chen Z. Li Y. Ma L. Zou Y. Wang X. (2020). Low density lipoprotein receptor related protein 6 (LRP6) protects heart against oxidative stress by the crosstalk of HSF1 and GSK3β. Redox Biol. 37, 101699. 10.1016/j.redox.2020.101699 32905882 PMC7486456

[B51] Xiao J. Xiao S. LuO J. Lu M. Lu M. Llu X. (2020). Advance in 3,4-seco-lupane type triterpenoids and their bioactivity. Chin. Traditional Herb. Drugs 52 (06), 1834–1843. 10.7501/j.issn.0253-2670.2021.06.033

[B52] Yin H. Yang Q. Cao Z. Li H. Yu Z. Zhang G. (2018). Activation of NLRX1-mediated autophagy accelerates the ototoxic potential of cisplatin in auditory cells. Toxicol. Appl. Pharmacol. 343, 16–28. 10.1016/j.taap.2018.02.007 29454061

[B53] Yu X. Man R. Li Y. Yang Q. Li H. Yang H. (2019). Paeoniflorin protects spiral ganglion neurons from cisplatin-induced ototoxicity: possible relation to PINK1/BAD pathway. J. Cell. Mol. Med. 23 (8), 5098–5107. 10.1111/jcmm.14379 31207045 PMC6653418

[B54] Yuan H. Wang X. Hill K. Chen J. Lemasters J. Yang S.-M. (2015). Autophagy attenuates noise-induced hearing loss by reducing oxidative stress. Antioxidants and Redox Signal. 22 (15), 1308–1324. 10.1089/ars.2014.6004 PMC4410759 25694169

[B55] Yun S.-E. Choi B.-B. r. Nam S.-H. Kim G.-C. (2023). Antimicrobial effects of edible mixed herbal extracts on oral microorganisms: an *in vitro* study. Medicina 59 (10), 1771. 10.3390/medicina59101771 37893489 PMC10608150

[B56] Zhao Y. Wang X. Chen C. Shi K. Li J. Du R. (2021). Protective effects of 3,4-seco-lupane triterpenes from food raw materials of the leaves of eleutherococcus senticosus and eleutherococcus sessiliflorus on arrhythmia induced by barium chloride. Chem. and Biodivers. 18 (4), e2001021. 10.1002/cbdv.202001021 33615691

[B57] Zhu J. Tian Z. Li Y. Hua X. Zhang D. Li J. (2019). ATG7 promotes bladder cancer invasion via autophagy-mediated increased ARHGDIB mRNA stability. Adv. Sci. 6 (8), 1801927. 10.1002/advs.201801927 PMC6468970 31016112

