# Supplementary material for: The chiisanoside derivatives present in the leaves of Acanthopanax sessiliflorus activate autophagy through the LRP6/GSK3β axis and thereafter inhibit oxidative stress, thereby counteracting cisplatin-induced ototoxicity
Source: Front Pharmacol. 2025 Jan 15;15:1518810. doi: 10.3389/fphar.2024.1518810 (PMC11774919; doi:10.3389/fphar.2024.1518810)
Supplement: Supplementary file 1 [file DataSheet2.pdf]

Bax·(Cell Repeat) 21 kDa

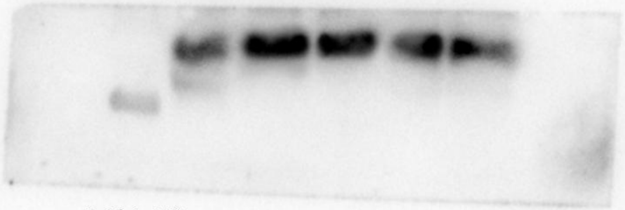

Compound 19(μM) – – 6.25 12.5 25

CDDP 50μM – + + + +

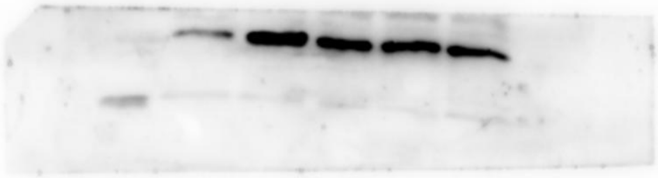

Compound 19(μM) – – 6.25 12.5 25

CDDP 50μM – + + + +

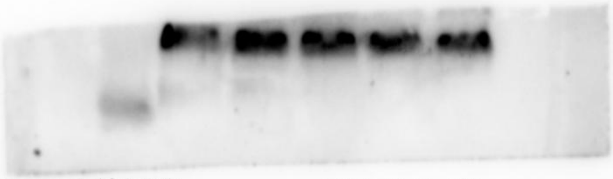

Compound 19(μM) – – 6.25 12.5 25

CDDP 50μM – + + + +

Bcl-2·(Cell Repeat) 26 kDa

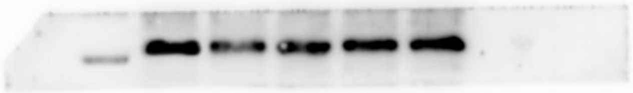

Compound 19(μM) – – 6.25 12.5 25

CDDP 50μM – + + + +

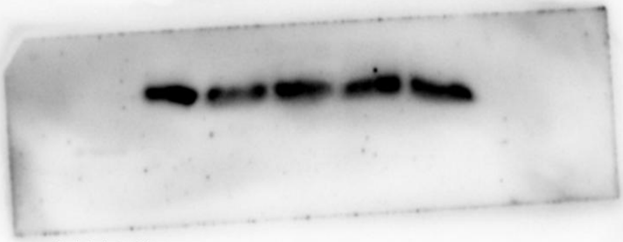

Compound 19(μM) – – 6.25 12.5 25

CDDP 50μM – + + + +

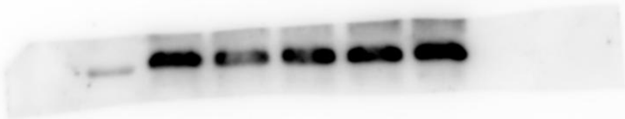

Compound 19(μM) – – 6.25 12.5 25

CDDP 50μM – + + + +

Cleaved-caspase3(Cell Repeat) 17 kDa

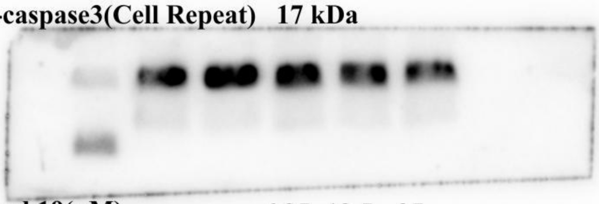

Compound 19(μM) – – 6.25 12.5 25

CDDP 50μM – + + + +

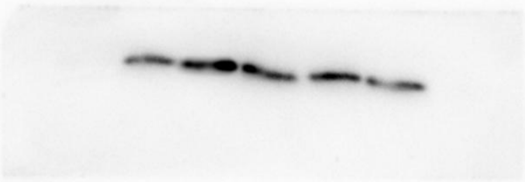

Compound 19(μM) – – 6.25 12.5 25

CDDP 50μM – + + + +

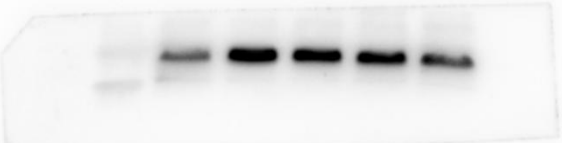

Compound 19(μM) – – 6.25 12.5 25

CDDP 50μM – + + + +

Caspase3(Cell Repeat) 35 kDa

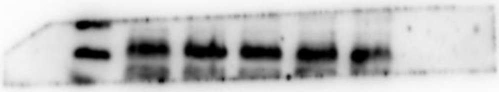

Compound 19(μM) – – 6.25 12.5 25

CDDP 50μM – + + + +

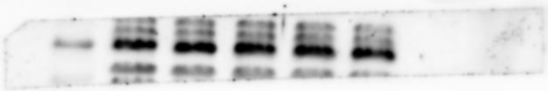

Compound 19(μM) – – 6.25 12.5 25

CDDP 50μM – + + + +

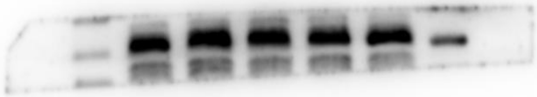

Compound 19(μM) – – 6.25 12.5 25

CDDP 50μM – + + + +

β-Actin(Cell Repeat) 42 kDa

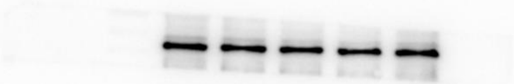

Compound 19(μM) – – 6.25 12.5 25

CDDP 50μM – + + + +

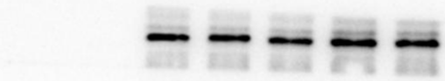

Compound 19(μM) – – 6.25 12.5 25

CDDP 50μM – + + + +

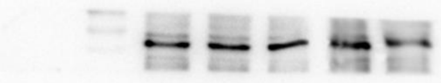

Compound 19(μM) – – 6.25 12.5 25

CDDP 50μM – + + + +

Atg 5·(Cell Repeat) 32 kDa

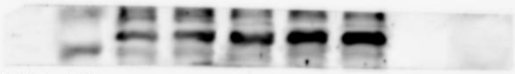

Compound 19(μM) – – 6.25 12.5 25

CDDP 50μM – + + + +

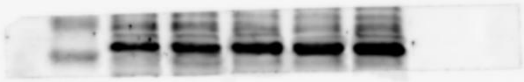

Compound 19(μM) – – 6.25 12.5 25

CDDP 50μM – + + + +

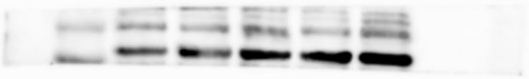

Compound 19(μM) – – 6.25 12.5 25

CDDP 50μM – + + + +

Atg 7·(Cell Repeat)   78 kDa

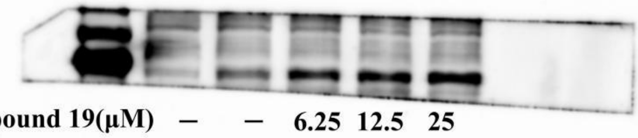

Compound 19(μM) – – 6.25 12.5 25

CDDP 50μM – + + + +

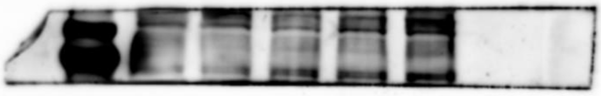

Compound 19(μM) – – 6.25 12.5 25

CDDP 50μM – + + + +

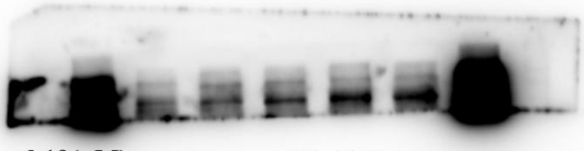

Compound 19(μM) – – 6.25 12.5 25

CDDP 50μM – + + + +

LC3(Cell Repeat) 14-16 kDa

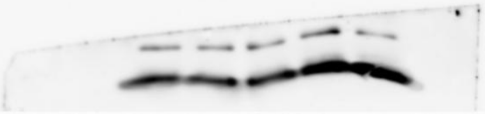

Compound 19(μM) – – 6.25 12.5 25

CDDP 50μM – + + + +

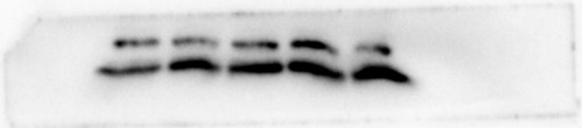

Compound 19(μM) – – 6.25 12.5 25

CDDP 50μM – + + + +

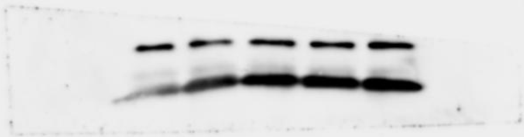

Compound 19(μM) – – 6.25 12.5 25

CDDP 50μM – + + + +

P62(Cell Repeat)   62 kDa

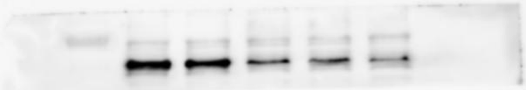

Compound 19(μM)   –   –   6.25   12.5   25

CDDP 50μM   –   +   +   +   +

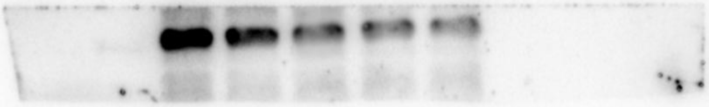

Compound 19(μM)   –   –   6.25   12.5   25

CDDP 50μM   –   +   +   +   +

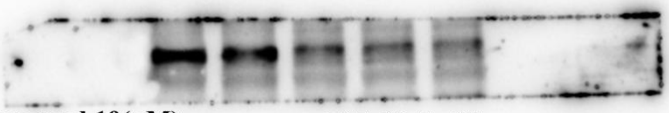

Compound 19(μM)   –   –   6.25   12.5   25

CDDP 50μM   –   +   +   +   +

β-Actin(Cell Repeat) 42 kDa

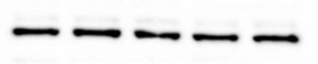

Compound 19(μM) – – 6.25 12.5 25

CDDP 50μM – + + + +

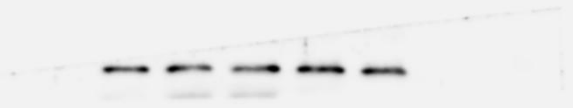

Compound 19(μM) – – 6.25 12.5 25

CDDP 50μM – + + + +

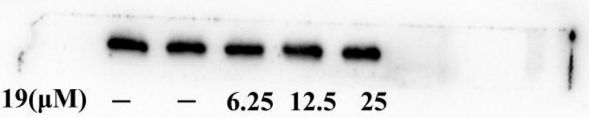

Compound 19(μM) – – 6.25 12.5 25

CDDP 50μM – + + + +

Bax(Cell Repeat) 21 kDa

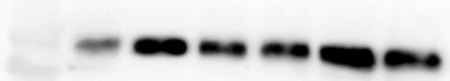

Compound 19(μM)	−	−	25	−	−	25
CDDP 50μM	−	+	+	−	+	+
3-MA	−	−	−	+	+	+

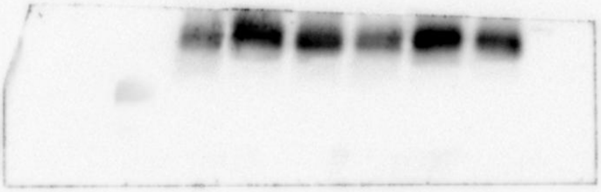

Compound 19(μM)	−	−	25	−	−	25
CDDP 50μM	−	+	+	−	+	+
3-MA	−	−	−	+	+	+

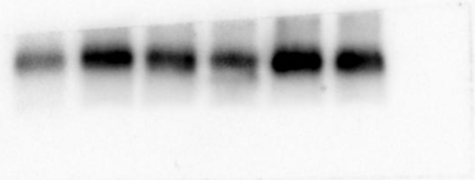

Compound 19(μM)	−	−	25	−	−	25
CDDP 50μM	−	+	+	−	+	+
3-MA	−	−	−	+	+	+

Bcl-2(Cell Repeat) 26 kDa

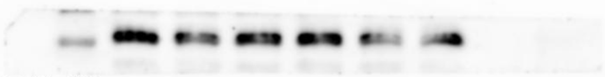

Compound 19(μM)	–	–	25	–	–	25
CDDP 50μM	–	+	+	–	+	+
3-MA	–	–	–	+	+	+

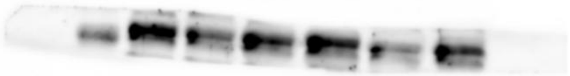

Compound 19(μM)	–	–	25	–	–	25
CDDP 50μM	–	+	+	–	+	+
3-MA	–	–	–	+	+	+

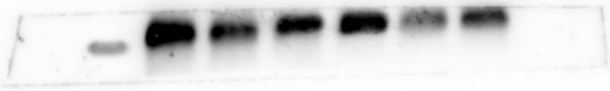

Compound 19(μM)	–	–	25	–	–	25
CDDP 50μM	–	+	+	–	+	+
3-MA	–	–	–	+	+	+

Cleaved-caspase3(Cell Repeat) 17 kDa

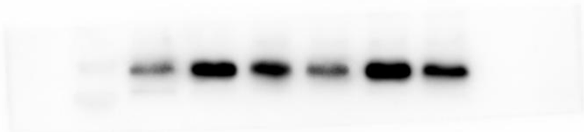

Compound 19(μM)	–	–	25	–	–	25
CDDP 50μM	–	+	+	–	+	+
3-MA	–	–	–	+	+	+

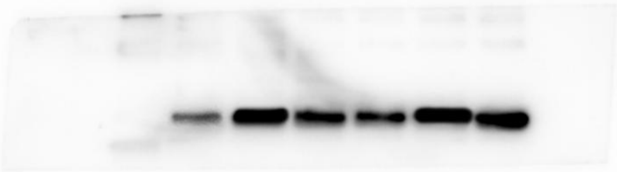

Compound 19(μM)	–	–	25	–	–	25
CDDP 50μM	–	+	+	–	+	+
3-MA	–	–	–	+	+	+

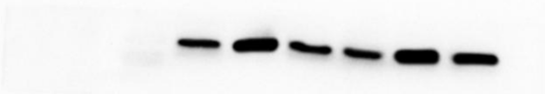

Compound 19(μM)	–	–	25	–	–	25
CDDP 50μM	–	+	+	–	+	+
3-MA	–	–	–	+	+	+

Caspase3(Cell Repeat) 35 kDa

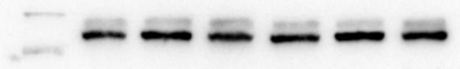

Compound 19(μM)	–	–	25	–	–	25
CDDP 50μM	–	+	+	–	+	+
3-MA	–	–	–	+	+	+

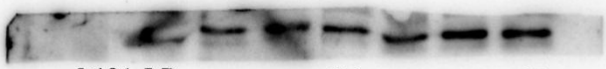

Compound 19(μM)	–	–	25	–	–	25
CDDP 50μM	–	+	+	–	+	+
3-MA	–	–	–	+	+	+

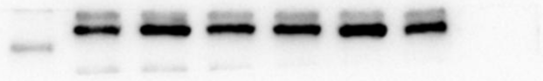

Compound 19(μM)	–	–	25	–	–	25
CDDP 50μM	–	+	+	–	+	+
3-MA	–	–	–	+	+	+

LC3(Cell Repeat) 14-16 kDa

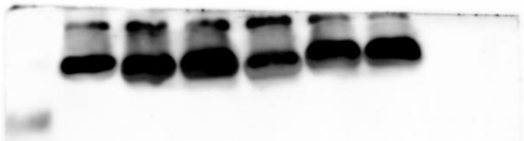

Compound 19(μM)	–	–	25	–	–	25
CDDP 50μM	–	+	+	–	+	+
3-MA	–	–	–	+	+	+

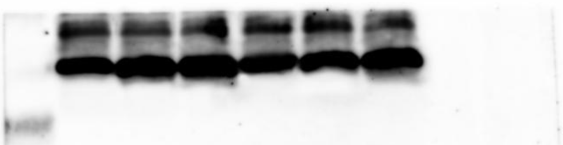

Compound 19(μM)	–	–	25	–	–	25
CDDP 50μM	–	+	+	–	+	+
3-MA	–	–	–	+	+	+

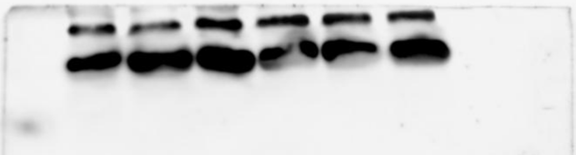

Compound 19(μM)	–	–	25	–	–	25
CDDP 50μM	–	+	+	–	+	+
3-MA	–	–	–	+	+	+

P62(Cell Repeat) 62 kDa

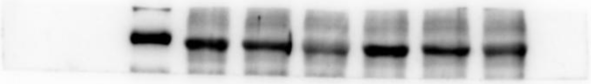

Compound 19(μM) – – 25 – – 25

CDDP 50μM – + + – + +

3-MA – – – + + +

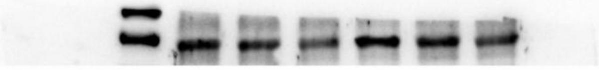

Compound 19(μM) – – 25 – – 25

CDDP 50μM – + + – + +

3-MA – – – + + +

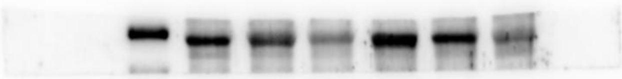

Compound 19(μM) – – 25 – – 25

CDDP 50μM – + + – + +

3-MA – – – + + +

β-Actin(Cell Repeat) 42 kDa

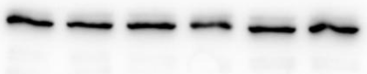

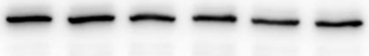

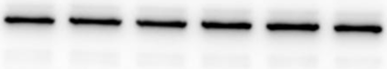

LRP6(Cell Repeat) 179 kDa

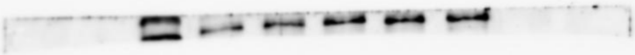

Compound 19(μM) – – 6.25 12.5 25

CDDP 50μM – + + + +

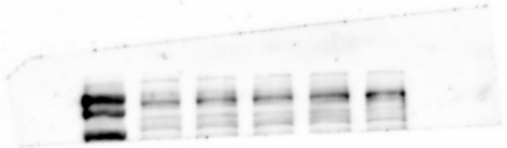

Compound 19(μM) – – 6.25 12.5 25

CDDP 50μM – + + + +

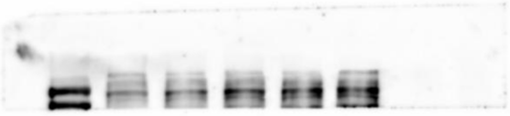

Compound 19(μM) – – 6.25 12.5 25

CDDP 50μM – + + + +

GSK3β(Cell Repeat) 47 kDa

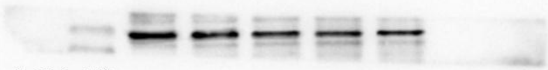

Compound 19(μM) – – 6.25 12.5 25

CDDP 50μM – + + + +

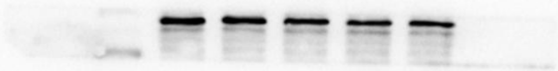

Compound 19(μM) – – 6.25 12.5 25

CDDP 50μM – + + + +

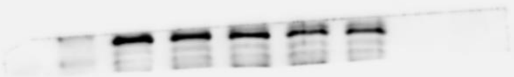

Compound 19(μM) – – 6.25 12.5 25

CDDP 50μM – + + + +

P-GSK3β(Cell Repeat) 47 kDa

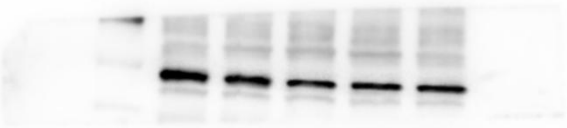

Compound 19(μM) – – 6.25 12.5 25

CDDP 50μM – + + + +

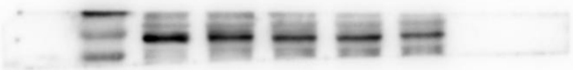

Compound 19(μM) – – 6.25 12.5 25

CDDP 50μM – + + + +

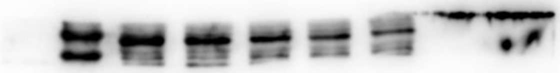

Compound 19(μM) – – 6.25 12.5 25

CDDP 50μM – + + + +

β-Actin(Cell Repeat)   42 kDa

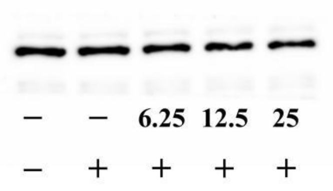

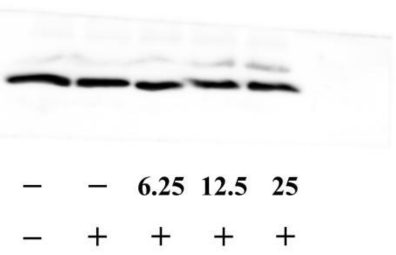

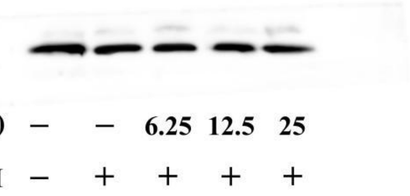

LRP6(Cell Repeat)   179 kDa

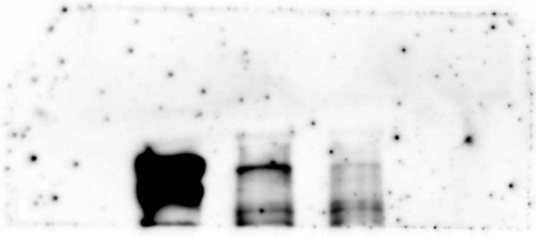

NC
Sh-LRP6

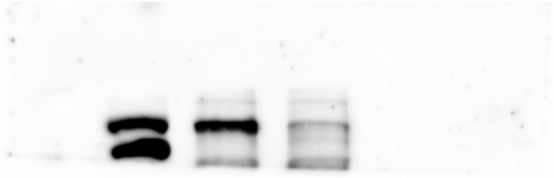

NC
Sh-LRP6

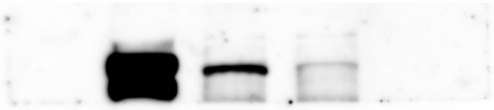

NC
Sh-LRP6

β-Actin(Cell Repeat)   42 kDa

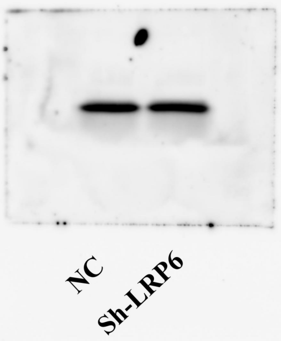

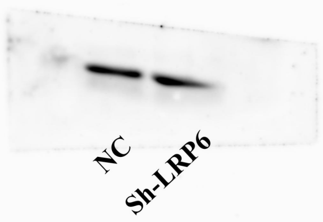

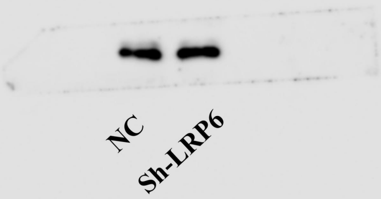

LRP6(Cell Repeat) 179 kDa

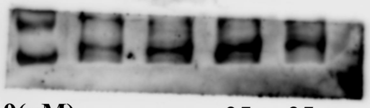

Compound 19(μM)	–	–	25	25
CDDP 50μM	–	+	+	+
Sh-LRP6	–	–	–	+

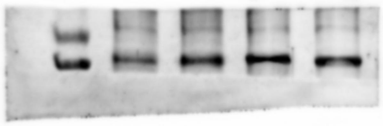

Compound 19(μM)	–	–	25	25
CDDP 50μM	–	+	+	+
Sh-LRP6	–	–	–	+

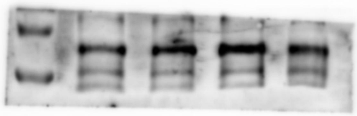

Compound 19(μM)	–	–	25	25
CDDP 50μM	–	+	+	+
Sh-LRP6	–	–	–	+

P-GSK3β(Cell Repeat)   47 kDa

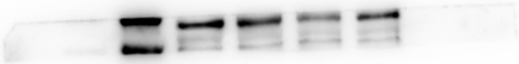

Compound 19(μM)	−	−	25	25
CDDP 50μM	−	+	+	+
Sh-LRP6	−	−	−	+

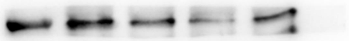

Compound 19(μM)	−	−	25	25
CDDP 50μM	−	+	+	+
Sh-LRP6	−	−	−	+

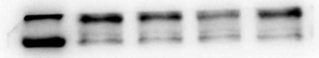

Compound 19(μM)	−	−	25	25
CDDP 50μM	−	+	+	+
Sh-LRP6	−	−	−	+

GSK3β(Cell Repeat) 47 kDa

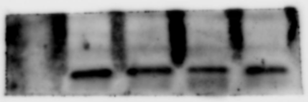

Compound 19(μM)	–	–	25	25
CDDP 50μM	–	+	+	+
Sh-LRP6	–	–	–	+

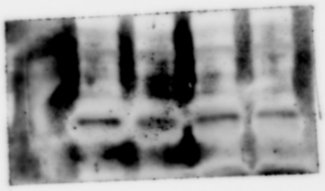

Compound 19(μM)	–	–	25	25
CDDP 50μM	–	+	+	+
Sh-LRP6	–	–	–	+

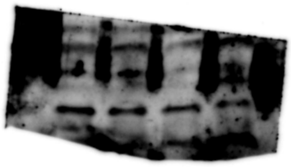

Compound 19(μM)	–	–	25	25
CDDP 50μM	–	+	+	+
Sh-LRP6	–	–	–	+

P62(Cell Repeat) 62 kDa

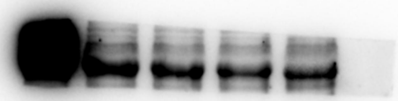

Compound 19(μM)	–	–	25	25
CDDP 50μM	–	+	+	+
Sh-LRP6	–	–	–	+

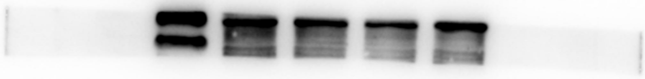

Compound 19(μM)	–	–	25	25
CDDP 50μM	–	+	+	+
Sh-LRP6	–	–	–	+

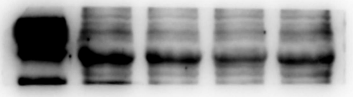

Compound 19(μM)	–	–	25	25
CDDP 50μM	–	+	+	+
Sh-LRP6	–	–	–	+

LC3(Cell Repeat) 14-16 kDa

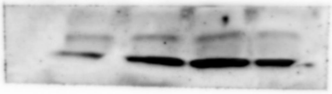

Compound 19(μM)	–	–	25	25
CDDP 50μM	–	+	+	+
Sh-LRP6	–	–	–	+

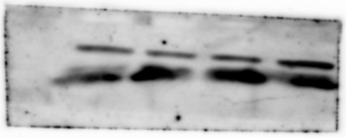

Compound 19(μM)	–	–	25	25
CDDP 50μM	–	+	+	+
Sh-LRP6	–	–	–	+

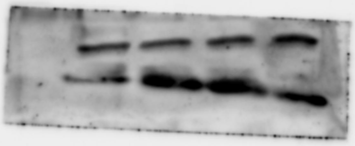

Compound 19(μM)	–	–	25	25
CDDP 50μM	–	+	+	+
Sh-LRP6	–	–	–	+

Bax(Cell Repeat) 21 kDa

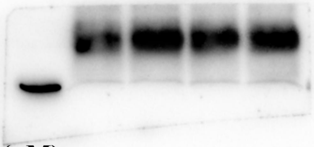

Compound 19(μM)	–	–	25	25
CDDP 50μM	–	+	+	+
Sh-LRP6	–	–	–	+

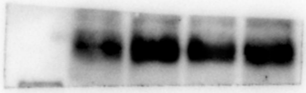

Compound 19(μM)	–	–	25	25
CDDP 50μM	–	+	+	+
Sh-LRP6	–	–	–	+

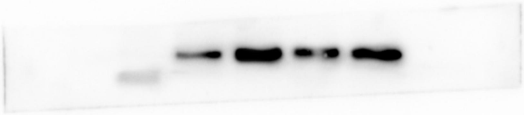

Compound 19(μM)	–	–	25	25
CDDP 50μM	–	+	+	+
Sh-LRP6	–	–	–	+

Bcl-2(Cell Repeat) 26 kDa

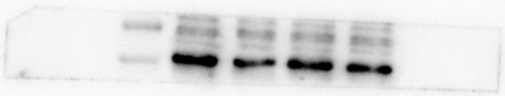

Compound 19(μM)	–	–	25	25
CDDP 50μM	–	+	+	+
Sh-LRP6	–	–	–	+

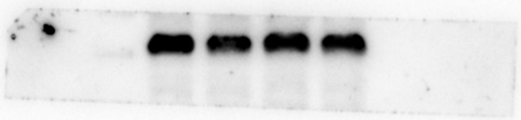

Compound 19(μM)	–	–	25	25
CDDP 50μM	–	+	+	+
Sh-LRP6	–	–	–	+

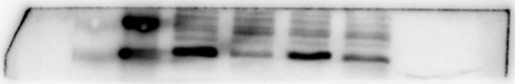

Compound 19(μM)	–	–	25	25
CDDP 50μM	–	+	+	+
Sh-LRP6	–	–	–	+

β-Actin(Cell Repeat) 42 kDa

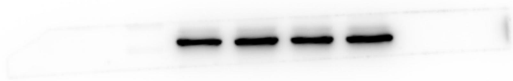

Compound 19(μM) – – 25 25

CDDP 50μM – + + +

Sh-LRP6 – – – +

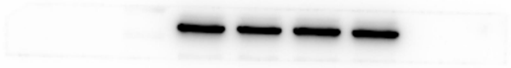

Compound 19(μM) – – 25 25

CDDP 50μM – + + +

Sh-LRP6 – – – +

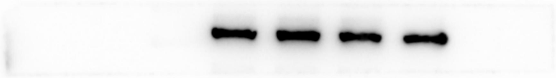

Compound 19(μM) – – 25 25

CDDP 50μM – + + +

Sh-LRP6 – – – +

P62 (Cell Repeat) 62 kDa

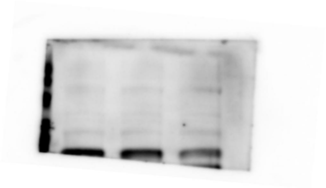

Compound 19(μM)	–	25	25
CDDP 50μM	+	+	+
LiCl	–	–	+

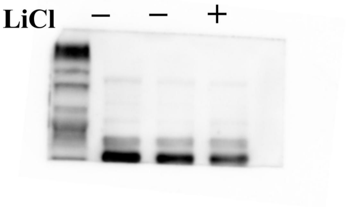

Compound 19(μM)	–	25	25
CDDP 50μM	+	+	+
LiCl	–	–	+

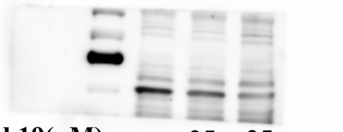

Compound 19(μM)	–	25	25
CDDP 50μM	+	+	+
LiCl	–	–	+

LC3 (Cell Repeat) 14-16 kDa

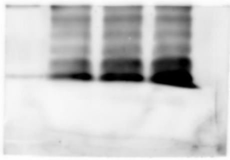

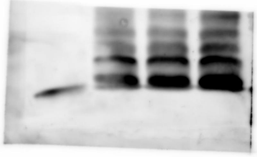

Compound 19(μM) – 25 25

CDDP 50μM + + +

LiCl – – +

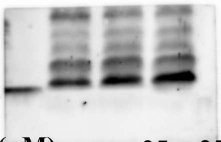

Compound 19(μM) – 25 25

CDDP 50μM + + +

LiCl – – +

β-Actin(Cell Repeat)   42 kDa

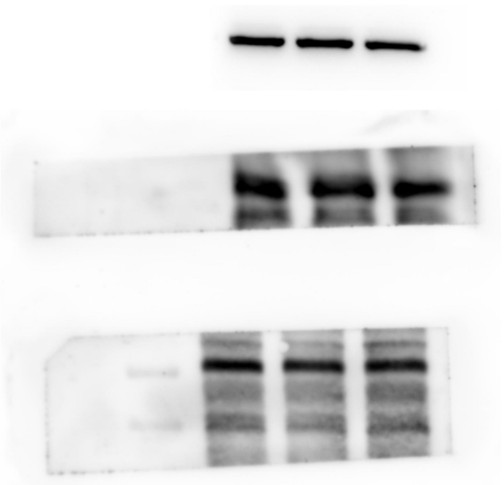

Compound 19(μM)	−	25	25
CDDP 50μM	+	+	+
LiCl	−	−	+

Bax·(Mouse Repeat) 21 kDa

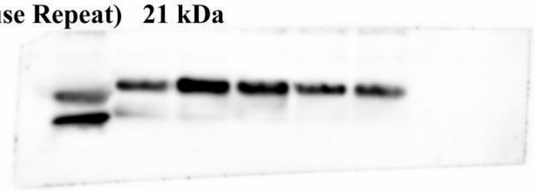

Compound 19(mg/kg) – – 10 20 40

CDDP 5mg/kg – + + + +

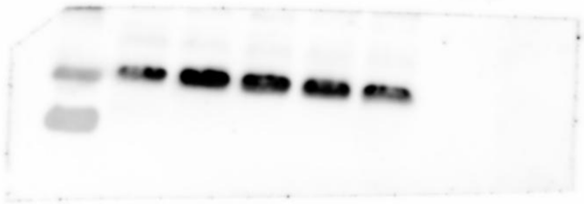

Compound 19(mg/kg) – – 10 20 40

CDDP 5mg/kg – + + + +

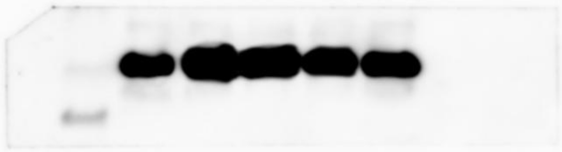

Compound 19(mg/kg) – – 10 20 40

CDDP 5mg/kg – + + + +

Bcl-2·(Mouse Repeat) 26 kDa

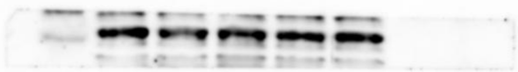

Compound 19(mg/kg) – – 10 20 40

CDDP 5mg/kg – + + + +

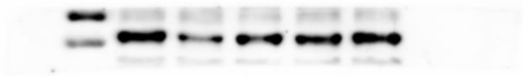

Compound 19(mg/kg) – – 10 20 40

CDDP 5mg/kg – + + + +

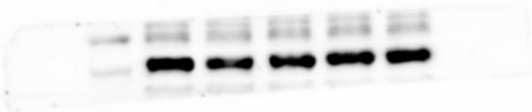

Compound 19(mg/kg) – – 10 20 40

CDDP 5mg/kg – + + + +

Cleaved-caspase3(Mouse Repeat) 17 kDa

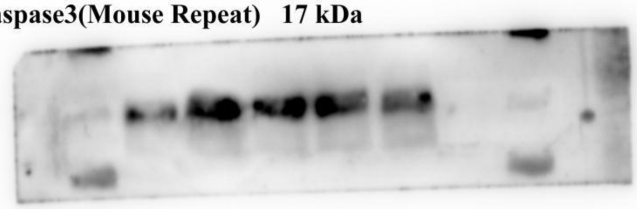

Compound 19(mg/kg) – – 10 20 40

CDDP 5mg/kg – + + + +

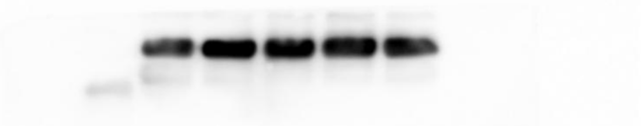

Compound 19(mg/kg) – – 10 20 40

CDDP 5mg/kg – + + + +

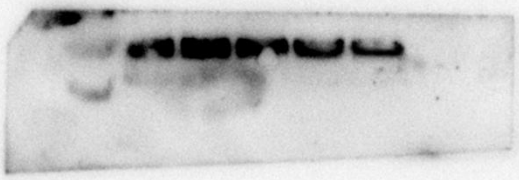

Compound 19(mg/kg) – – 10 20 40

CDDP 5mg/kg – + + + +

Caspase3(Mouse Repeat)   35 kDa

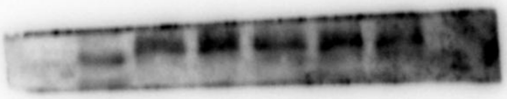

Compound 19(mg/kg) – – 10 20 40

CDDP 5mg/kg – + + + +

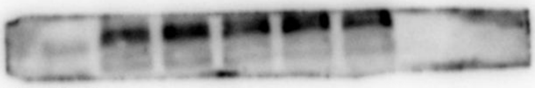

Compound 19(mg/kg) – – 10 20 40

CDDP 5mg/kg – + + + +

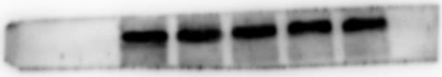

Compound 19(mg/kg) – – 10 20 40

CDDP 5mg/kg – + + + +

β-Actin(Mouse Repeat) 42 kDa

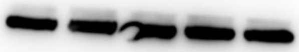

Compound 19(mg/kg) – – 10 20 40

CDDP 5mg/kg – + + + +

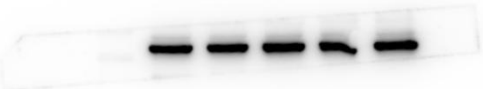

Compound 19(mg/kg) – – 10 20 40

CDDP 5mg/kg – + + + +

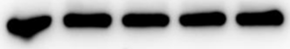

Compound 19(mg/kg) – – 10 20 40

CDDP 5mg/kg – + + + +

Atg 5·(Mouse Repeat) 32 kDa

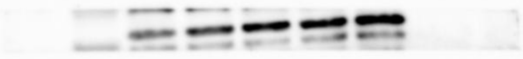

Compound 19(mg/kg) – – 10 20 40

CDDP 5mg/kg – + + + +

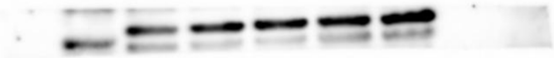

Compound 19(mg/kg) – – 10 20 40

CDDP 5mg/kg – + + + +

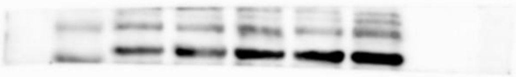

Compound 19(mg/kg) – – 10 20 40

CDDP 5mg/kg – + + + +

Atg 7·(Mouse Repeat) 78 kDa

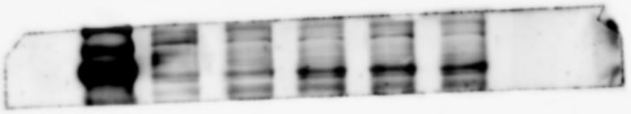

Compound 19(mg/kg) – – 10 20 40

CDDP 5mg/kg – + + + +

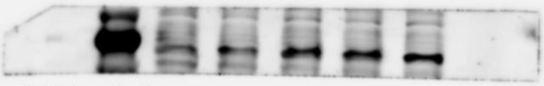

Compound 19(mg/kg) – – 10 20 40

CDDP 5mg/kg – + + + +

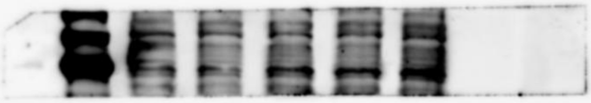

Compound 19(mg/kg) – – 10 20 40

CDDP 5mg/kg – + + + +

LC3(Mouse Repeat)   14-16 kDa

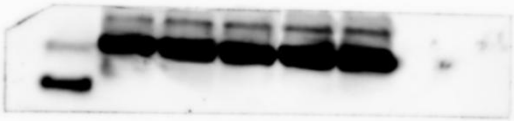

Compound 19(mg/kg) – – 10 20 40

CDDP 5mg/kg – + + + +

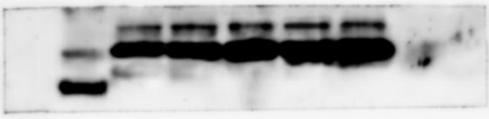

Compound 19(mg/kg) – – 10 20 40

CDDP 5mg/kg – + + + +

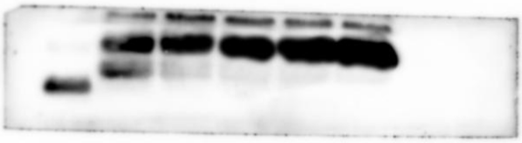

Compound 19(mg/kg) – – 10 20 40

CDDP 5mg/kg – + + + +

P62(Mouse Repeat) 62 kDa

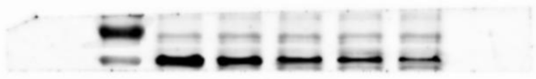

Compound 19(mg/kg)	–	–	10	20	40
CDDP 5mg/kg	–	+	+	+	+

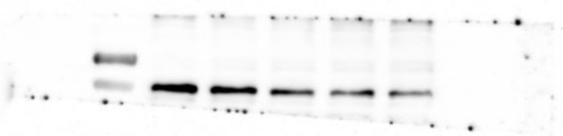

Compound 19(mg/kg)	–	–	10	20	40
CDDP 5mg/kg	–	+	+	+	+

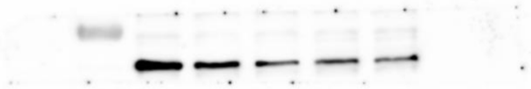

Compound 19(mg/kg)	–	–	10	20	40
CDDP 5mg/kg	–	+	+	+	+

β-Actin(Mouse Repeat) 42 kDa

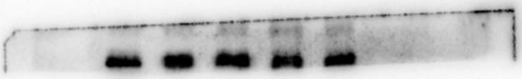

Compound 19(mg/kg) – – 10 20 40

CDDP 5mg/kg – + + + +

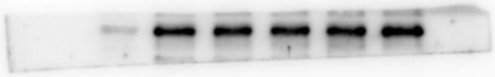

Compound 19(mg/kg) – – 10 20 40

CDDP 5mg/kg – + + + +

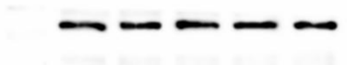

Compound 19(mg/kg) – – 10 20 40

CDDP 5mg/kg – + + + +

LRP6(Mouse Repeat) 179 kDa

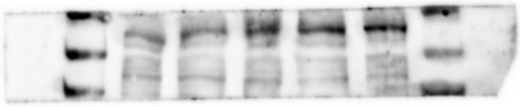

Compound 19(mg/kg) – – 10 20 40

CDDP 5mg/kg – + + + +

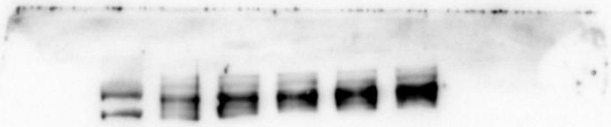

Compound 19(mg/kg) – – 10 20 40

CDDP 5mg/kg – + + + +

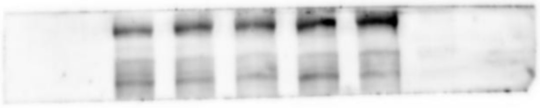

Compound 19(mg/kg) – – 10 20 40

CDDP 5mg/kg – + + + +

GSK3β(Mouse Repeat)   47 kDa

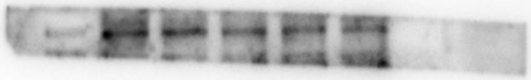

Compound 19(mg/kg) – – 10 20 40

CDDP 5mg/kg – + + + +

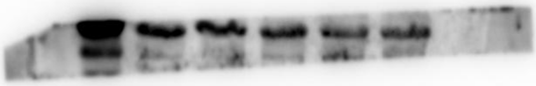

Compound 19(mg/kg) – – 10 20 40

CDDP 5mg/kg – + + + +

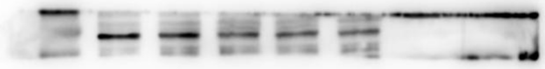

Compound 19(mg/kg) – – 10 20 40

CDDP 5mg/kg – + + + +

P-GSK3β(Mouse Repeat)   47 kDa

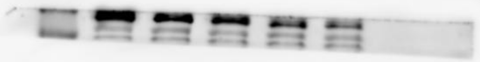

Compound 19(mg/kg) – – 10 20 40

CDDP 5mg/kg – + + + +

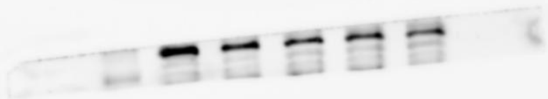

Compound 19(mg/kg) – – 10 20 40

CDDP 5mg/kg – + + + +

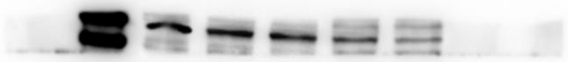

Compound 19(mg/kg) – – 10 20 40

CDDP 5mg/kg – + + + +

β-Actin(Mouse Repeat) 42 kDa

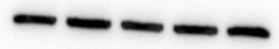

Compound 19(mg/kg) – – 10 20 40

CDDP 5mg/kg – + + + +

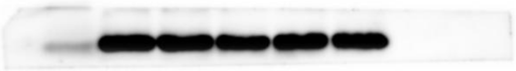

Compound 19(mg/kg) – – 10 20 40

CDDP 5mg/kg – + + + +

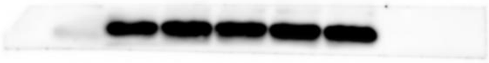

Compound 19(mg/kg) – – 10 20 40

CDDP 5mg/kg – + + + +
