# Supplementary material for: The chiisanoside derivatives present in the leaves of Acanthopanax sessiliflorus activate autophagy through the LRP6/GSK3β axis and thereafter inhibit oxidative stress, thereby counteracting cisplatin-induced ototoxicity
Source: Front Pharmacol. 2025 Jan 15;15:1518810. doi: 10.3389/fphar.2024.1518810 (PMC11774919; doi:10.3389/fphar.2024.1518810)
Supplement: Supplementary file 2 [file DataSheet1.docx]

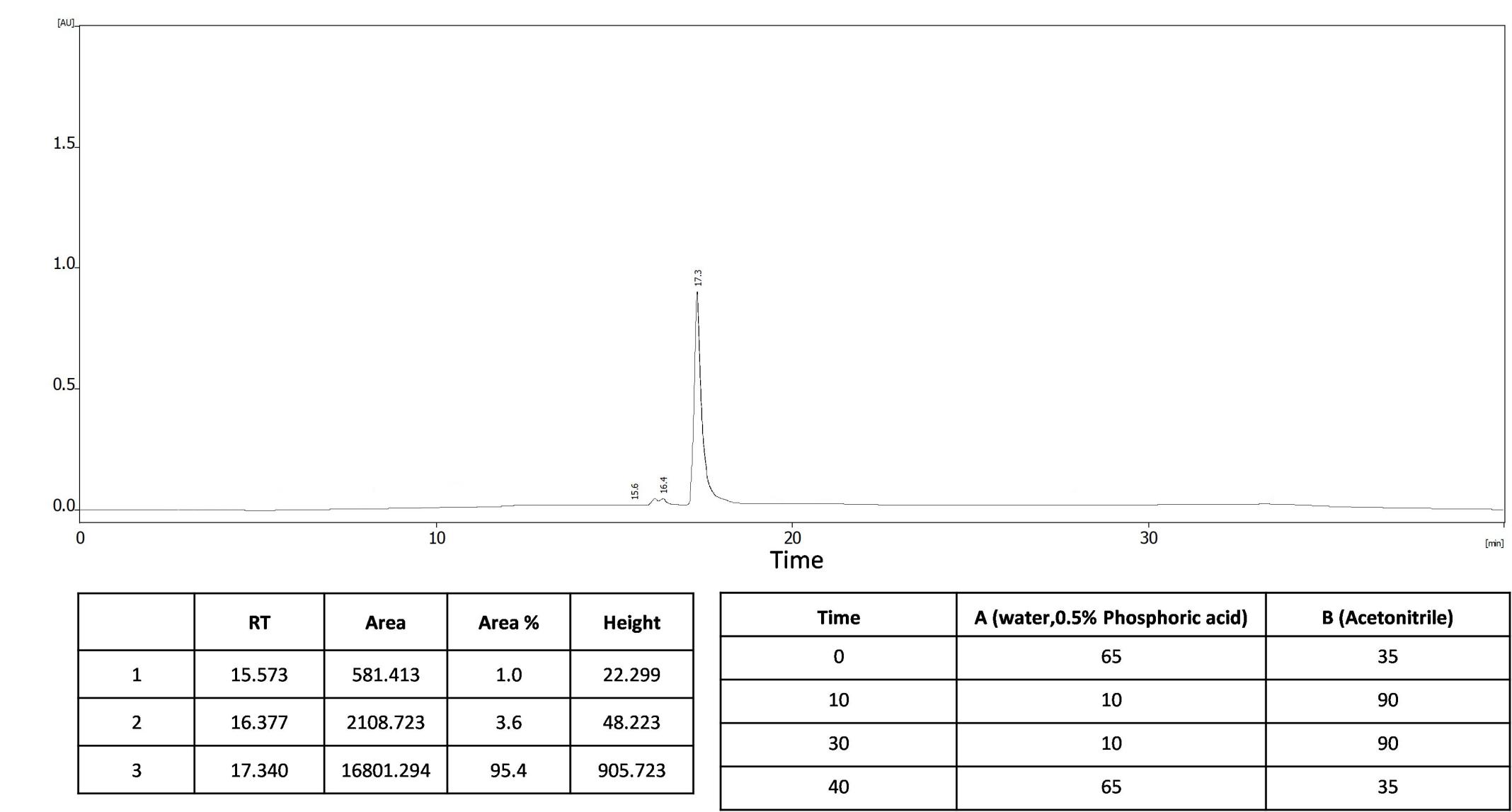
Figure S1. *HPLC tracing of compound* ***19****.*


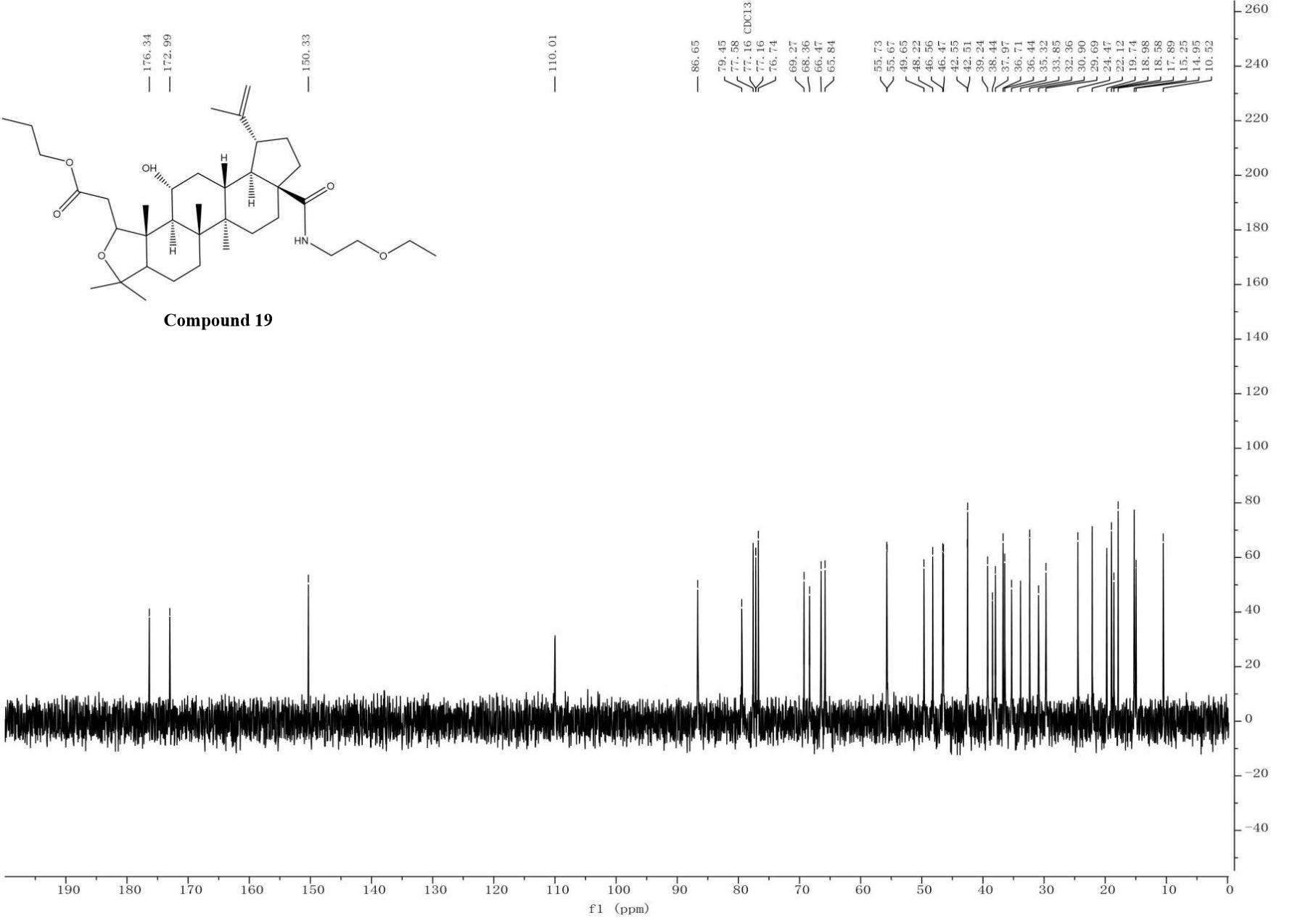


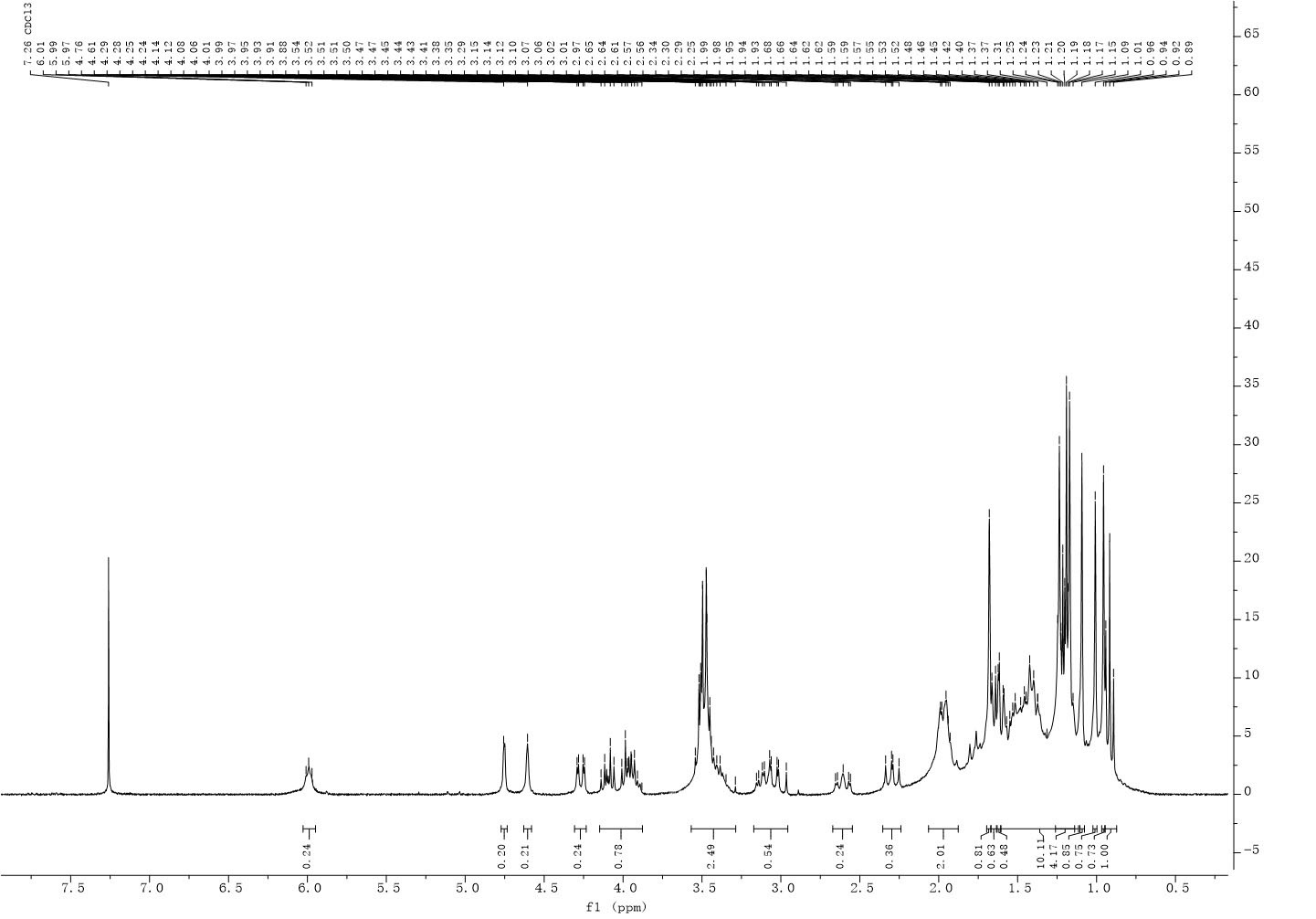


Figures S2. *^13^C and ^1^H NMR of compound* ***19.***

Table.S1 Primer sequences for qPCR analysis

|  | Forward Primer，5’-3’ | Reverse Primer，5’-3’ |
| --- | --- | --- |
| GAPDH | AGGAGCGAGACCCCACTAACATCAA | ATGGGGGCATCGGCAGAAGGGGCGG |
| LC3 | GACCGCTGTAAGGAGGTGC | CTTGACCAACTCGCTCATGTTA |
| P62 | GACTACGACTTGTGTAGCGTC | AGTGTCCGTGTTTCACCTTCC |
| ATG5 | CAACTTGTTTCACGCTATATCAGG | CACTTTGTCAGTTACCAA CGTCA |
| ATG7 | TAATGTCCTTCCCGTCAGCCT | TCATGTCCCAGATCTCAGCAG |
| GSK3β | CCACTCAAGAACTGTCAAGTAACC | CCACGGTCTCCAGCATTAGTA |
| LRP6 | CACTTACTTCCCTGCAATTTTGAACC | TGGCCTGTAGGTATGACCTATG |

Table.S2 DEGs of autophagy signaling pathway in HEI-OC1 cells.

| gene symbol | MeanTPM (compound 19) | MeanTPM (CDDP) | log2 FC | Pvalue | regulated by compound 19 |
| --- | --- | --- | --- | --- | --- |
| Atg7 | 2.554643 | 0.4183755 | 2.61025142 | 0.000129371 | Up |
| Atg5 | 6.342896 | 0.698805 | 3.182179852 | 0.000237953 | Up |
| Sqstm1 | 0.134842 | 6.680636 | -5.630643619 | 5.00E-07 | Down |
| Map1lc3b | 12.2724035 | 0.1445635 | 6.407570672 | 1.62E-05 | Up |

Table.S3 DEGs of the LRP6/GSK3β signaling pathway in HEI-OC1 cells.

| gene symbol | MeanTPM (compound 19) | MeanTPM (CDDP) | log2 FC | Pvalue | regulated by compound 19 |
| --- | --- | --- | --- | --- | --- |
| Lrp6 | 18.3069495 | 2.198014 | 3.058118933 | 2.77E-19 | Up |
| Gsk3b | 1.0551215 | 7.972878 | -2.917691455 | 1.36E-05 | Down |


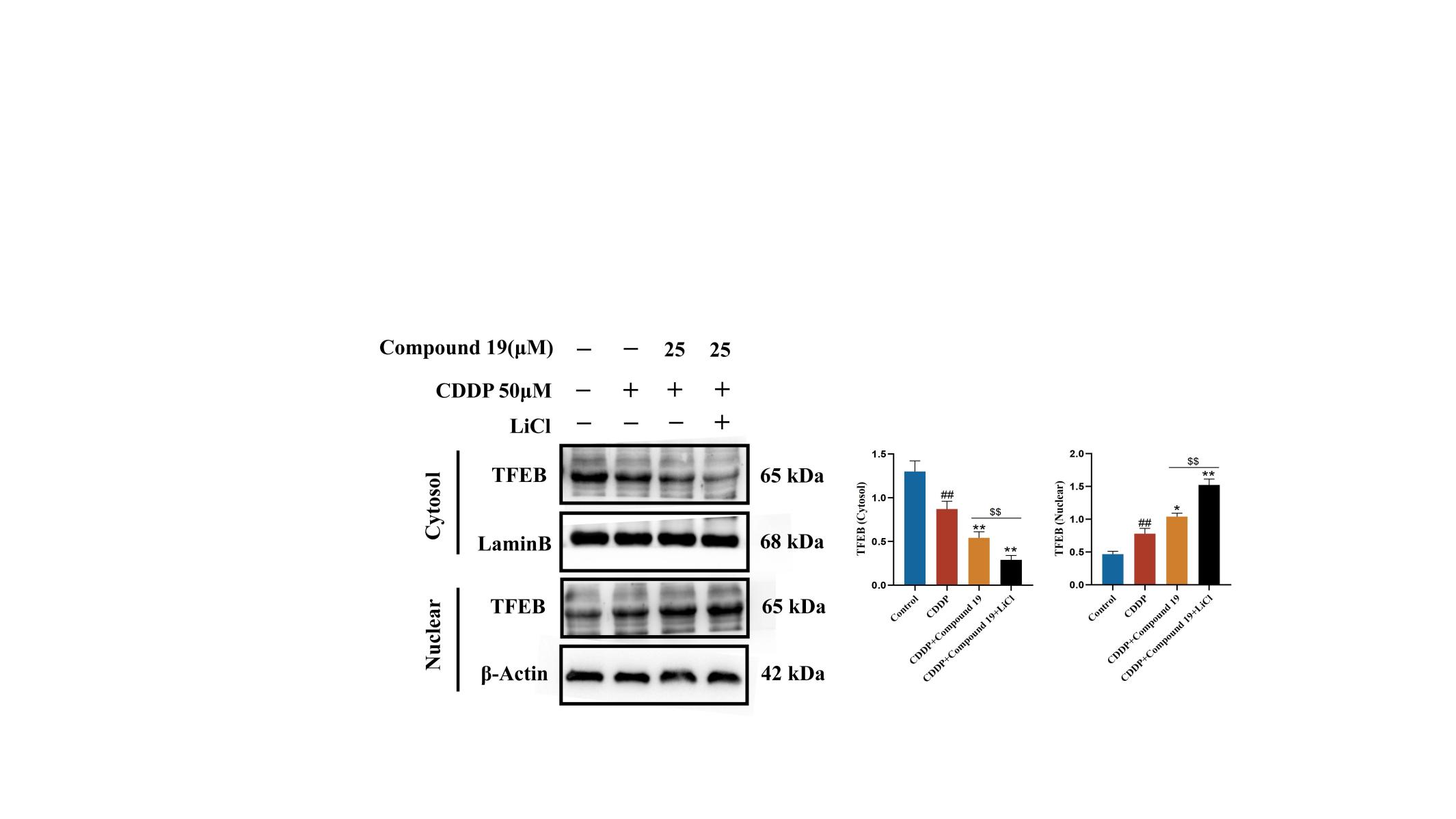


**Fig. S3. The effect of compound 19 on the expression of TFEB protein in HEI-OC1 cells induced by cisplatin under the action of LiCl.**

**Fig. S4.Weight value of mice in each group**
